# Identification of novel HIV-1 dependency factors in primary CCR4^+^CCR6^+^Th17 cells via a genome-wide transcriptional approach

**DOI:** 10.1186/s12977-015-0226-9

**Published:** 2015-12-10

**Authors:** Aurélie Cleret-Buhot, Yuwei Zhang, Delphine Planas, Jean-Philippe Goulet, Patricia Monteiro, Annie Gosselin, Vanessa Sue Wacleche, Cécile L. Tremblay, Mohammad-Ali Jenabian, Jean-Pierre Routy, Mohamed El-Far, Nicolas Chomont, Elias K. Haddad, Rafick-Pierre Sekaly, Petronela Ancuta

**Affiliations:** Department of Microbiology, Infectiology and Immunology, Faculty of Medicine, Université de Montréal, Montreal, QC Canada; CHUM-Research Centre, 900 rue Saint-Denis, Tour Viger, R09.416, Montreal, QUÉBEC H2X 0A9 Canada; Caprion, Montreal, QC Canada; Département des sciences biologiques, Université du Québec à Montréal, Montreal, QC Canada; Chronic Viral Illness Service, McGill University Health Centre, Montreal, QC Canada; Research Institute, McGill University Health Centre, Montreal, QC Canada; Division of Hematology, McGill University Health Centre, Montreal, QC Canada; Division of infectious Diseases and HIV Medicine, Drexel University, Philadelphia, PA USA; Center for AIDS Research, Case Western Reserve University, Cleveland, OH USA

**Keywords:** Human Th17 cells, HIV-1 dependency factors, TCR, NF-κB, MAP3K4, PTPN13

## Abstract

**Background:**

The HIV-1 infection is characterized by profound CD4^+^ T cell destruction and a marked Th17 dysfunction at the mucosal level. Viral suppressive antiretroviral therapy restores Th1 but not Th17 cells. Although several key HIV dependency factors (HDF) were identified in the past years via genome-wide siRNA screens in cell lines, molecular determinants of HIV permissiveness in primary Th17 cells remain to be elucidated.

**Results:**

In an effort to orient Th17-targeted reconstitution strategies, we investigated molecular mechanisms of HIV permissiveness in Th17 cells. Genome-wide transcriptional profiling in memory CD4^+^ T-cell subsets enriched in cells exhibiting Th17 (CCR4^+^CCR6^+^), Th1 (CXCR3^+^CCR6^−^), Th2 (CCR4^+^CCR6^−^), and Th1Th17 (CXCR3^+^CCR6^+^) features revealed remarkable transcriptional differences between Th17 and Th1 subsets. The HIV-DNA integration was superior in Th17 versus Th1 upon exposure to both wild-type and VSV-G-pseudotyped HIV; this indicates that post-entry mechanisms contribute to viral replication in Th17. Transcripts significantly enriched in Th17 versus Th1 were previously associated with the regulation of TCR signaling (ZAP-70, Lck, and CD96) and Th17 polarization (RORγt, ARNTL, PTPN13, and RUNX1). A meta-analysis using the *NCBI HIV Interaction Database* revealed a set of Th17-specific HIV dependency factors (HDFs): PARG, PAK2, KLF2, ITGB7, PTEN, ATG16L1, Alix/AIP1/PDCD6IP, LGALS3, JAK1, TRIM8, MALT1, FOXO3, ARNTL/BMAL1, ABCB1/MDR1, TNFSF13B/BAFF, and CDKN1B. Functional studies demonstrated an increased ability of Th17 versus Th1 cells to respond to TCR triggering in terms of NF-κB nuclear translocation/DNA-binding activity and proliferation. Finally, RNA interference studies identified MAP3K4 and PTPN13 as two novel Th17-specific HDFs.

**Conclusions:**

The transcriptional program of Th17 cells includes molecules regulating HIV replication at multiple post-entry steps that may represent potential targets for novel therapies aimed at protecting Th17 cells from infection and subsequent depletion in HIV-infected subjects.

**Electronic supplementary material:**

The online version of this article (doi:10.1186/s12977-015-0226-9) contains supplementary material, which is available to authorized users.

## Background

The Th17 cells represent a distinct lineage of CD4^+^ T-cells characterized by the expression of specific transcription factors (e.g., RORγt, RORA, and STAT3) and cytokines (e.g., IL-17A, IL-17F, IL-21, IL-22, IL-26, IL-8, and CCL20) [1–5]. Th17 cells represent unique players in immunity against pathogens at mucosal barrier surfaces where they orchestrate the functionality of epithelial cells, neutrophils, and B cells [3, 6, 7, 8, 9, 10]. Recruitment of Th17 cells into mucosal sites is mediated in part by the homing receptor CCR6/CCL20, with CCR6 being a well-established Th17 surface marker [11, 12]. Other homing receptors, such as CCR4 and CXCR3, distinguish between Th17 subsets with distinct antigenic specificity and effector cytokine expression: CCR4^+^CCR6^+^Th17 and CXCR3^+^CCR6^+^Th1Th17 [13–15]. During chronic HIV/SIV infections, the depletion of Th17 cells from gut-associated lymphoid tissues (GALT) leads to dramatic alterations of the mucosal barrier integrity, alterations that cause microbial translocation, chronic immune activation, and disease progression [16–28]. Studies in SIV models demonstrated an inverse correlation between peak and set point viral loads as well as the preexisting mucosal Th17 pool [29]; this strengthens the concept that Th17 cells significantly contribute to anti-viral immunity at mucosal sites [30]. Studies in HIV-infected subjects demonstrated that the preservation of mucosal Th17 cells is associated with slow disease progression [31–36]. Despite the success of current antiretroviral therapies (ART) in reducing viral replication to undetectable plasma levels, the pool of Th17 cells is not fully restored at mucosal sites or in the peripheral blood of infected subjects [22, 31, 37, 38, 39]. Recent studies demonstrated that ART initiation during early but not late acute HIV infection preserves Th17 counts and their effector functions [40, 41]. However, early HIV diagnosis remains, however a challenge even in high income countries; this emphasizes the need for alternative strategies with the goal of Th17 preservation and/or restoration during chronic HIV infection.

The mechanisms underlying Th17 depletion during HIV/SIV infections include altered trafficking into mucosal sites [42, 43]; altered ratios between regulatory T-cells (Tregs) and Th17 cells [44, 45]; depletion of mucosal CD103^+^ dendritic cells (DC) [46], a subset involved in Th17 differentiation [47, 48]; limited IL-21 availability, a cytokine critical for Th17 survival [27]; and/or over expression of negative regulators of Th17 differentiation [49]. In addition, studies by our group and others provided evidence that infection *per se* contributes to the depletion of memory Th17 cells [37, 38, 50] and the paucity of naive-like Th17 precursors [39, 51]. Despite their massive depletion, fractions of Th17 cells are long lived [52–54] and likely contribute to HIV persistence under ART [55] (Wacleche, Ancuta et al, unpublished observations). Genome-wide RNA interference studies performed in distinct cell lines identified large sets of HIV dependency factors (HDFs) and revealed the molecular complexity of virus-host cell interactions [56–60]. Nevertheless, the molecular determinants of HIV permissiveness in primary Th17 cells are not fully understood. This knowledge is essential for designing novel targeted therapies aiming at limiting HIV replication and persistence specifically in Th17 cells.

In this study, we investigated transcriptional and functional differences between primary memory CD4^+^ T-cell subsets enriched in Th17 (CCR4^+^CCR6^+^) and Th1 (CXCR3^+^CCR6^−^) polarized cells, subsets that we previously reported to be permissive and resistant to infection with R5 or X4 HIV strains, respectively [37]. Our study revealed the existence of HDFs specifically expressed by Th17 cells that may be used as targets for novel therapeutic strategies aiming at limiting HIV replication and preserving the quality of Th17-mediated mucosal immunity in HIV-infected subjects.

## Results

### Identification of a molecular signature associated with HIV permissiveness in Th17 cells at entry and post-entry levels

**Fig. 1:**
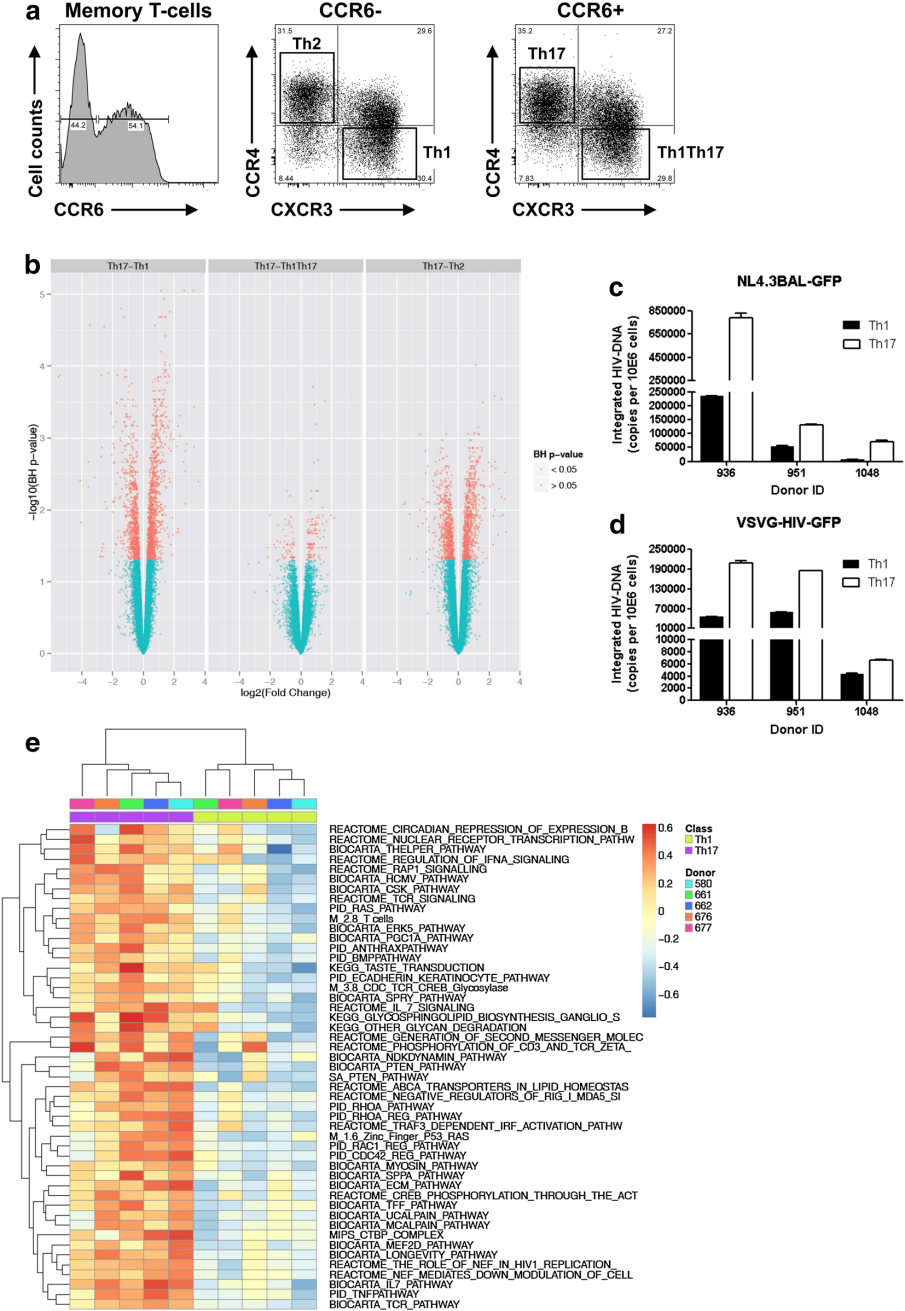
Identification of a molecular signature associated with HIV permissiveness in CCR4^+^CCR6^+^ Th17 cells. Total CD4^+^ T-cells were isolated from PBMCs of HIV-uninfected subjects by negative selection using magnetic beads. Cells were labeled with a cocktail of CD45RA, CD8, CD19, CD56, CCR4, CCR6, and CXCR3 Abs. **a** Shown is the gating strategy used for the FACS sorting of the following four memory (CD45RA^−^) CD4^+^ subsets lacking CD8, CD19, and CD56 expression: CXCR3^+^CCR4^−^CCR6^−^ (Th1), CXCR3^−^CCR4^+^CCR6^−^ (Th2), CXCR3^−^CCR4^+^CCR6^+^ (Th17), and CXCR3^+^CCR4^−^CCR6^+^ (Th1Th17). Shown are results from one donor representative of results generated with cells from >10 HIV-uninfected donors. **b** Highly pure matched Th1, Th2, Th17, and Th1Th17 subsets were sorted by FACS (n = 5) and stimulated via CD3/CD28 for 3 days. Total RNA was reverse transcribed into cDNA and hybridized onto the Human HT-12 v4 Expression BeadChip (Illumina) for genome-wide transcriptional profiling. One-way ANOVA analysis was performed to identify differentially expressed genes based on p value < 0.05 and fold change (FC, cut-off 1.3). Shown are volcano plots for all probes in each linear model with the FC on the x axis and the negative logarithm of the adjusted p values adjusted for false discovery rate (FDR) on the *y* axis. *Red/green color* code is based on the 5 % FDR threshold. **c**, **d** Cell subsets were stimulated via CD3/CD28 for 3 days and exposed to replication competent NL4.3BAL-GFP **(c)** and single round VSVG-HIV-GFP strains **(d)**. Shown is real-time quantification of HIV-DNA integration at day three post-infection (mean ± SD of triplicate wells) in matched subsets isolated from n = 3 different donors. **e** Shown are the top 50 pathways up regulated in Th17 versus Th1 cells; heat map of the individual enrichment statistics (ES) from a gene set variation analysis (GSVA) for pathways differentially expressed between Th1 and Th17 cells with a FDR inferior to 1 %. Transcriptional profiles in **a**, **b**, and **e** were generated with cells stimulated via CD3/CD28 but unexposed to HIV

We previously demonstrated that subsets of memory CD4^+^ T-cells enriched in Th17 and Th1Th17 cells are highly permissive to R5 and X4 HIV infection; Th2-enriched fractions replicate X4 HIV only; while Th1-enriched fractions replicated R5 and X4 HIV at relatively low levels [37]. Except for Th2 cells that lack CCR5 expression, differences in HIV replication between Th17 and Th1 are not explained by differential expression of CCR5 or CXCR4 [37, 38]. To identify HIV-dependency factors (HDFs) in primary Th17 cells, we performed a genome-wide analysis of gene expression in memory CD4^+^ T-cell subsets enriched in Th1, Th2, Th17, and Th1Th17 cells sorted by FACS and stimulated by CD3/CD28 Abs, as previously described [37]. These subsets were identified based on the differential expression of the well-established surface markers CCR4, CCR6, and CXCR3, as previously described [13, 15, 37] and illustrated in Fig. 1a: Th1 (CXCR3^+^CCR4^−^CCR6^−^), Th2 (CXCR3^−^CCR4^+^CCR6^−^), Th17 (CXCR3^−^CCR4^+^CCR6^+^), and Th1Th17 (CXCR3^+^CCR4^−^CCR6^+^). Total mRNA extracted from each subset was hybridized onto the Illumina HumanHT-12 v4 Expression BeadChip (GEO access number GSE70396) and transcripts up- and down regulated in Th17 compared to Th1, Th2, or Th1Th17 were identified based on p values (p < 0.05) or adjusted p values (adj. p < 0.05) and fold change (FC) expression ratios (cut-off 1.3-fold) (Additional file 1: Table S1; Additional file 2: Table S2). The most robust differences in gene expression were observed between Th17 versus Th1 (Fig. 1b) with 1630 (p < 0.05) and 1,081 (adj. p < 0.05) up regulated and 1409 (p < 0.05) and 772 (adj. p < 0.05) down regulated probe sets (FC cut-off 1.3) (Additional file 1: Table S1; Additional file 2: Table S2; Additional file 3: Figure S1a). To orient our genome-wide search for HDFs, we investigated whether HIV permissiveness in Th17 versus Th1 was modulated at entry as opposed to post-entry levels. With this in mind, HIV-DNA integration was quantified in cells exposed to replication-competent R5 HIV (NL4.3BaL-GFP) or single-round VSV-G-pseudotyped HIV (VSVG-HIV-GFP) entering cells by endocytosis independently of CD4 and co-receptors [61]. Results in Fig. 1c, d reveal superior HIV-DNA integration in Th17 versus Th1 upon exposure to both NL4.3BAL-GFP and VSVG-HIV-GFP strains; this indicates that post-entry mechanisms contribute to HIV permissiveness in Th17 cells. This evidence led to the prediction that transcripts enriched in Th17 as compared to Th1 include HDFs acting at the post-entry level.

Top differentially expressed transcripts (adj. p < 0.05, FC > 2.5) in Th17 versus Th1 included well-established markers for Th17 (i.e., IL-22, CCR6, KLRB1, IL-17F, CCL20, RORC, IL-26, and PPARG) and Th1 (i.e., IFN-γ, CCL5, CCL4L2, CCL3, CCL3L3, and CXCR3); this provides a first validation for our transcriptional studies (Tables 1, 2). The list of top up regulated genes also included transcripts that likely represent new functional markers for Th17 cells: PTPN13 (APO-1/CD95 (Fas)-Associated Phosphatase) [62], CHN1, GPR56, LGMN, CTSH, KLF2, CACNA1l, RARRES3, Ly9, TNFSF13B, NFL2, PI16, MXD4, HPGD, SNX29, P2RY5, ZNF381, LIME1, MAP3K4, CD96, GPR15, and GLIPR1 (Table 1). Importantly, NFIL3, a documented inhibitor of Th17 polarization in mice [63], was found down regulated in human Th17 versus Th1 (Table 2); this suggests a conserved mechanism involved in the regulation of Th17 polarization in humans and mice.

**Table 1:** Top up regulated transcripts in CCR4^+^CCR6^+^ Th17 versus CXCR3^+^CCR6^−^ Th1

Symbol	p	adj. p	FC	Gene definition
IL-22	1.0E−04	4.0E−03	11.9	Interleukin 22
CCR6	1.7E−06	3.7E−04	9.7	Chemokine (C–C motif) receptor 6
PTPN13	1.5E−09	8.9E−06	9.6	Protein tyrosine phosphatase, non-receptor type 13 (APO-1/CD95 (Fas)-associated phosphatase)
KLRB1	1.5E−05	1.2E−03	7.0	Killer cell lectin-like receptor subfamily B, member 1
IL-17F	5.9E−05	2.9E−03	6.6	Interleukin 17F
CCL20	3.2E−04	8.2E−03	6.6	Chemokine (C–C motif) ligand 20
RORC	2.1E−05	1.5E−03	6.6	RAR-related orphan receptor C, transcript variant
IL-26	9.3E−06	9.3E−04	5.1	Interleukin 26
GPR56	1.4E−03	2.0E−02	4.9	G protein-coupled receptor 56, transcript variant 2
LGMN	1.6E−05	1.3E−03	4.5	Legumain, transcript variant 2
CTSH	1.5E−03	2.1E−02	4.4	Cathepsin H, transcript variant 1
KLF2	1.7E−03	2.2E−02	3.8	Kruppel-like factor 2 (lung)
RARRES3	4.9E−03	4.3E−02	3.5	Retinoic acid receptor responder 3
Ly9	8.6E−06	8.8E−04	3.5	Lymphocyte antigen 9, transcript variant 2
TNFSF13B	2.1E−05	1.5E−03	3.5	Tumor necrosis factor superfamily, member 13b
NLF2	2.8E−03	3.1E−02	3.4	Nuclear localized factor 2
PI16	6.1E−09	1.7E−05	3.3	Peptidase inhibitor 16
MXD4	1.1E−07	9.8E−05	3.3	MAX dimerization protein 4
HPGD	4.1E−06	5.9E−04	3.3	Hydroxyprostaglandin dehydrogenase 15-(NAD)
SNX29	1.9E−05	1.4E−03	3.2	Sorting nexin 29
TRIB2	2.5E−07	1.4E−04	3.2	Tribbles homolog 2
P2RY5	2.3E−07	1.3E−04	3.1	Purinergic receptor P2Y, G-protein coupled, 5
ZNF381	6.4E−06	7.3E−04	3.0	Zinc finger protein 831
LIME1	1.1E−03	1.8E−02	2.9	Lck interacting transmembrane adaptor 1
MAP3K4	4.3E−07	1.7E−04	2.9	Mitogen-activated protein kinase kinase kinase 4
CD96	1.0E−05	9.8E−04	2.8	CD96 molecule, transcript variant 1
GPR15	1.7E−06	3.7E−04	2.8	G protein-coupled receptor 15
PPARG	3.2E−06	5.1E−04	2.7	Peroxisome proliferator-activated receptor gamma
GLIPR1	6.4E−06	7.3E−04	2.6	GLI pathogenesis-related 1
CD52	3.1E−04	3.2E−03	2.3	CD52 molecule
ARNTL	3.4E−04	8.4E−03	2.2	Aryl hydrocarbon receptor nuclear translocator-like
FOXO3	5.0E−03	4.3E−02	1.7	Forkhead box O3 (FOXO3), transcript variant 2, mRNA

*p* p value*; adj. p* adjusted p value*, FC* fold change

**Table 2:** Top down regulated transcripts in CCR4^+^CCR6^+^ Th17 *vs.* CXCR3^+^CCR6^−^ Th1

Symbol	p	adj. p	FC	Definition
IL-9	2.7E−07	1.4E−04	−46.7	Interleukin 9
GZMK	9.7E−05	3.9E−03	−17.1	Granzyme K (granzyme 3; tryptase II)
IFN-γ	8.5E−04	1.5E−02	−16.7	Interferon gamma
CCL5	3.8E−05	2.2E−03	−11.9	Chemokine (C–C motif) ligand 5
GZMH	1.4E−04	4.8E−03	−11.7	Granzyme H (cathepsin G-like 2, protein h-CCPX)
IL-3	2.2E−08	2.7E−05	−11.2	Interleukin 3 (colony-stimulating factor, multiple)
NKG7	8.4E−05	3.6E−03	−9.1	Natural killer cell group 7 sequence
CCL4L2	5.1E−03	4.4E−02	−7.0	Chemokine (C–C motif) ligand 4-like 2
CCL3	5.3E−03	4.5E−02	−6.8	Chemokine (C–C motif) ligand 3
EOMES	2.3E−07	1.3E−04	−6.6	Eomesodermin homolog (Xenopus laevis)
CCL3L1	3.9E−03	3.8E−02	−6.5	Chemokine (C–C motif) ligand 3-like 1
CCL3L3	5.2E−03	4.5E−02	−6.3	Chemokine (C–C motif) ligand 3-like 3
NAPSB	2.7E−03	3.0E−02	−6.1	Homo sapiens napsin B aspartic peptidase pseudogene (NAPSB)
BATF3	2.5E−08	2.8E−05	−5.9	Basic leucine zipper transcription factor, ATF-like 3
NAPSA	3.1E−04	7.9E−03	−5.6	Napsin A aspartic peptidase
CXCR3	4.2E−04	9.7E−03	−5.2	Chemokine (C–X–C motif) receptor 3
MATK	1.9E−07	1.2E−04	−4.9	Megakaryocyte-associated tyrosine kinase, transcript variant 3
OSM	2.5E−06	4.7E−04	−4.3	Oncostatin M
IRF8	2.5E−05	1.7E−03	−4.2	Interferon regulatory factor 8
MAOA	3.0E−04	7.8E−03	−4.0	Monoamine oxidase A, nuclear gene encoding mitochondrial protein
DNAJC12	5.1E−04	1.1E−02	−3.9	DnaJ (Hsp40) homolog. subfamily C, member 12
MT1 J	6.1E−06	7.1E−04	−3.9	Metallothionein 1G
NPSR1	1.5E−05	1.2E−03	−3.9	Neuropeptide S receptor 1, transcript variant 1
ATP8B4	4.0E−05	2.2E−03	−3.8	ATPase. class I, type 8B. member 4
TIMD4	1.8E−05	1.4E−03	−3.8	T-cell immunoglobulin and mucin domain containing 4
LTA	3.8E−05	2.2E−3	−3.7	Lymphotoxin alpha (TNF superfamily. member 1)
SERPINB6	2.1E−07	1.3E−04	−3.2	Serpin peptidase inhibitor, clade B (ovalbumin). member 6
PTK2	5.1E−03	4.4E−02	−2.7	PTK2 protein tyrosine kinase 2, transcript variant 2
CCL17	2.3E−05	1.6E−03	−2.6	Chemokine (C–C motif) ligand 17
NFIL3	3.4E−03	3.5E−02	−2.5	Nuclear factor interleukin 3 regulated

*p p* value, *adj. p* adjusted *p* value*; FC*, fold change

*Gene Set Variation Analysis* (GSVA) of differentially expressed genes (p < 0.05; FC cut-off 1.3) identified canonical pathways (C2) enriched in Th17 versus Th1, including those linked to circadian repression of expression by REV-ERBα, nuclear receptor transcription, T helper differentiation, CSK signaling, TCR signaling, Ras, anthrax, IL-7 signaling, phosphorylation of CD3 and TCR zeta, PTEN, ABCA transporters in lipid homeostasis, RhoA, longevity pathway, MEF2D signaling, the role of Nef in HIV replication, and TNF signaling (Fig. 1e). The GSVA also identified pathways down regulated in Th17 versus Th1, including pathways linked to metal ion SLC transporters, zinc transporters, STEM, glucose transport, extension of telomeres, protein synthesis as well as transcription initiation and termination (Additional file 3: Figure S1b; Additional file 4: Table S3). These results reveal overrepresentation of specific transcripts and cellular functions in Th17 versus Th1, with pathways enriched in Th17 cells likely being essential for both Th17 polarization and HIV permissiveness.

**Fig. 2:**
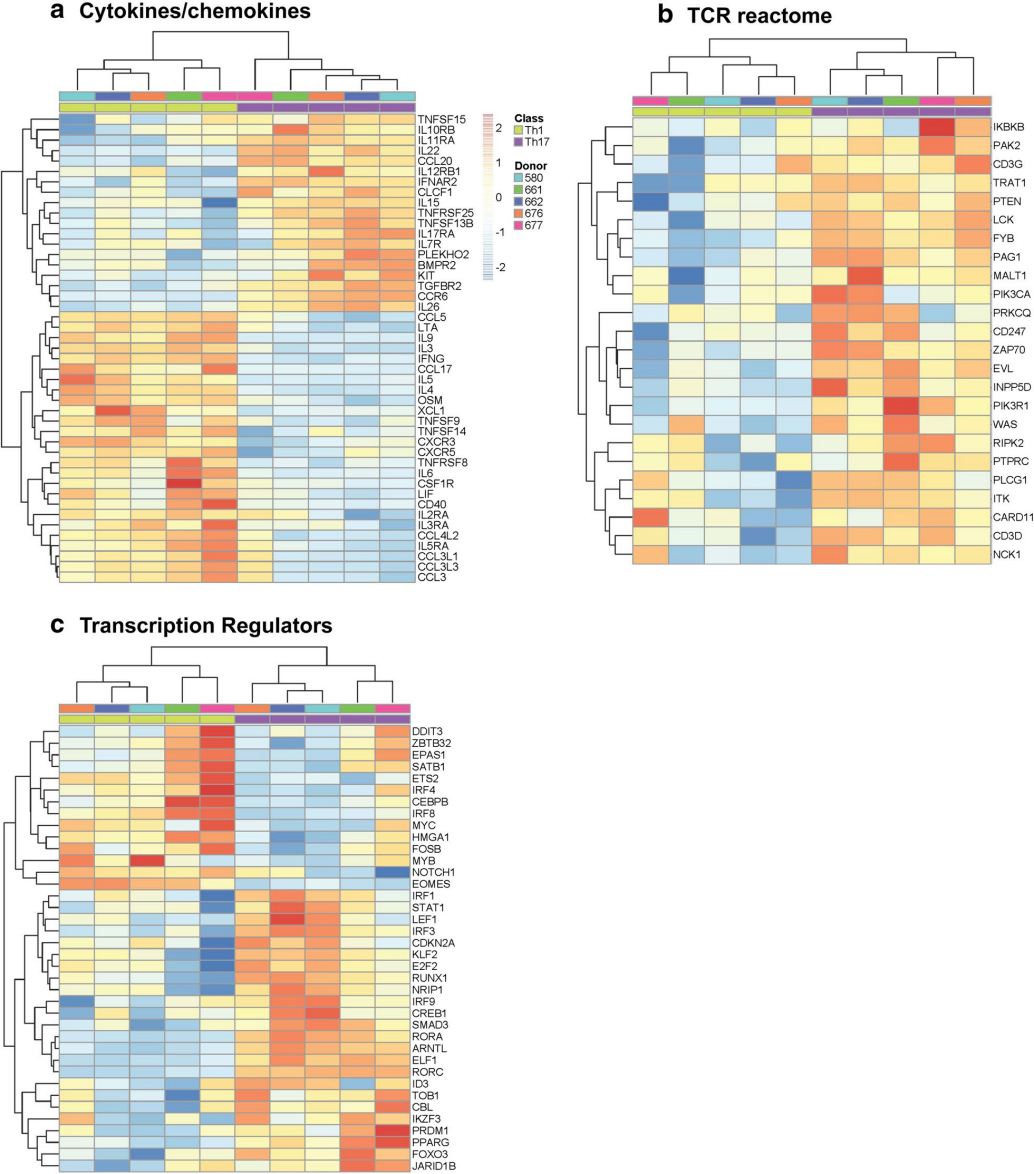
*Gene Ontology* classification of differentially expressed genes in CCR4^+^CCR6^+^ Th17 versus CXCR3^+^CCR6^−^ Th1 cells. Shown are heatmaps of differentially expressed genes (p < 0.05, FC cut-off 1.3) selected for their biological functions as follows: **a** Cytokines (KEGG), **b** TCR signalling (reactome), and **c** transcription regulators (ingenuity). *Coloring* of the cells is scaled by the z score of each microarray probe individually. Results correspond to matched Th17 and Th1 subsets from n = 5 different HIV-uninfected donors isolated and stimulated by CD3/CD28 Abs for 3 days as described in Fig. 1

Consistent with the GSVA results (Fig. 1e), *Gene Ontology* classification of differentially expressed genes (p < 0.05; FC cut-off 1.3) revealed transcripts related to different biological functions including: cytokines/chemokines, TCR, and transcription regulators (Fig. 2a–c). In the cytokines/chemokines category, genes up regulated in Th17 versus Th1 included known Th17-specific cytokines (IL-22, CCL20, IL-26) and chemokine receptors (CCR6), as well as TNFSF15 (TNF superfamily), TNFRSF25 (TNF receptor superfamily), TNFSF13B, IL-10RB, IL-11RA, CLCF1 (cardiotrophin-like cytokine factor), IL-15, IL-17RA, IL-7R, and TGFBR2 (TGF-β receptor) transcripts (Fig. 2a). Genes up regulated in Th1 versus Th17 included the Th1 marker CXCR3, IFNG, several CCR5 binding ckemokines (CCL5, CCL4L2, CCL3L1, CCL3L3, CCL3), and also transcripts for LTA (lymphotoxin α), IL-9, IL-3, CCL17, IL-5, IL-4, XCL1, TNFSF9, TNFSF14, CXCR5, TNFRSF8, IL-6, CSF1R, LIF, IL-2RA, IL3RA, and IL15RA (Fig. 2a). Despite the fact that IL-4 and IL-5 transcripts were found up regulated in Th1 versus Th17 cells, levels of their expression in Th1 compared to Th2 cells were significantly lower (data not shown). Such false positive signals are expected in high throughput screenings and therefore the requirement for subsequent validations for any important hit is mandatory. Transcripts related to the TCR signaling cascade such as IKBKB (inhibitor of NF-κB kinase beta) [64], PAK2 (p21-activated kinase 2) [65], CD3G, TRAT1 (T-cell receptor associated transmembrane adaptor 1), PTEN (phosphatase and tensin homolog) [66], Lck [67], FYB, PAG1, MALT1 [68, 69], PIK3CA, PRKCQ, CD247/CD3ε, ZAP70 [70], EVL, INPP5D, PIK3R1, WAS, RIPK2, PTPRC, PLCG1, ITK, CARD11, CD3D, and NCK1, were selectively enriched in Th17 versus Th1 (Fig. 2b). Of note, PAK2 [65] and ZAP70 [71] were previously linked to efficient HIV replication in T cells. Finally, major differences between Th17 and Th1 were observed for the expression of Transcription regulators. In addition to known transcription factors involved in the regulation of Th17 polarization (RORA, RORC, RUNX1) [72] and Th1 (Eomes) [73], other transcripts such as IRF1, STAT1, IRF3, KLF2, E2F2, IRF9, SMAD3, ARNTL, PPARG, and FOXO3 were found up regulated, while IRF4, IRF8, MYC, and Notch1 were down regulated in Th17 versus Th1 (Fig. 2c).

Similar to GSVA and GO, Gene Set Enrichment Analysis (GSEA) [74] identified top genes linked to canonical pathways (TCR signaling: PAK2, Lck, ZAP-70, CD96), transcription factors (RORC, RUNX1), and biological processes (stress activated protein kinase signaling: MAP4K1, MAP3K4, MAP3K5; protein amino acid dephosphorylation: PTPN12, PTPN13, PTPN22) as being up regulated in Th17 versus Th1 cells (Additional file 5: Figure S2).

**Fig. 3:**
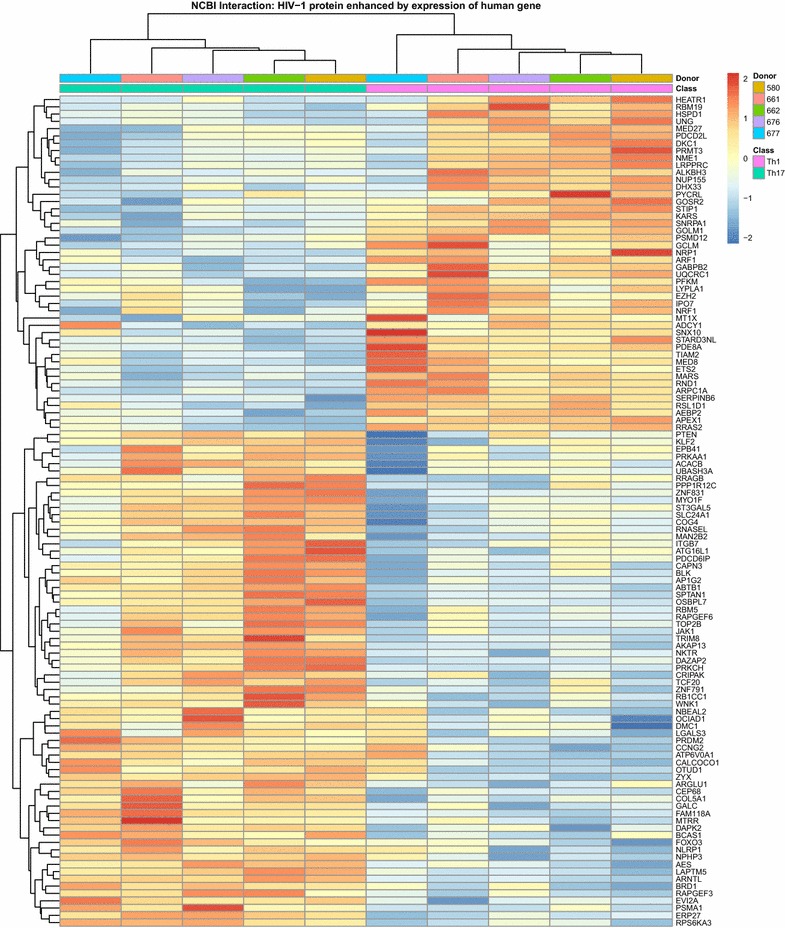
Meta-analysis using the NCBI HIV interaction database for the identification of HIV-1 proteins enhanced by expression of human genes enriched in Th17 cells. Differentially expressed genes between Th17 and Th1 subsets (p < 0.05, FC cut-off 1.3) were matched to the lists of human genes known to interact with HIV-1 proteins. Shown are significant probes with the smallest p value for each overlapping gene. Heatmap cells are scaled by the expression level z scores for each probe individually

We also performed a meta-analysis to identify transcripts enriched in Th17 versus Th1 that overlapped with those listed on the National Center for Biotechnology Information (NCBI) interaction database in the categories “HIV-1 proteins enhanced by expression of human genes” (Fig. 3) and “HIV-1 proteins interact with” (Additional file 6: Figure S3). Such transcripts included PTEN (a negative regulator of the mTOR pathway [75]), KLF2 [76], ITGB7 (a gut-homing molecule and an alternative HIV-binding receptor [77]), ATG16L1 (a regulator of autophagy, a pathway associated with HIV intracellular degradation [78, 79]), PDCD6IP (AIP1/Alix, a component of the HIV budding machinery [80]), JAK1 (a tyrosine kinase that phosphorylates STAT3 [60]), TRIM8 (a regulator of NF-κB and STAT3-dependent signaling cascades [81, 82] and a documented HDF [57]), LGALS3 (an HIV Tat-induced glycoprotein recently reported to promote HIV budding by association with Alix [79, 83]), FOXO3 [84], ARNTL [84] (Fig. 3), ABCB1 (a drug efflux pump associated with resistance to antiretroviral and anti-cancer drugs [85]), TNFSF13B/BAFF (a gene targeted by HIV for integration [86]), RUNX1 (a transcription factors involved in HIV latency [87]), PAK2 [65], and CDKN1B/p27kip1 (Additional file 6: Figure S3).

**Fig. 4:**
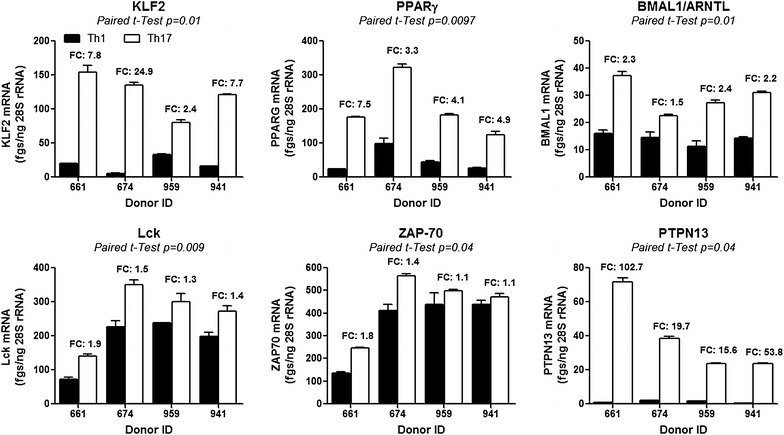
Validation by RT-PCR of superior KLF2, PPARγ, ARNTL, Lck, ZAP-70, and PTPN13 mRNA expression in CCR4^+^CCR6^+^ Th17 versus CXCR3^+^CCR6^−^ Th1 cells. Total RNA was extracted from Th17 and Th1 subsets isolated and stimulated via CD3/CD28 for 3 days as described in Fig. 1. Expression of KLF2, PPARγ, ARNTL, PTPN13, Lck, and ZAP-70 mRNA was quantified by SYBR green real time RT-PCR. Quantification was performed relative to a standard curve generated based on cDNA specific for each transcript. The expression of each gene was normalized to the 28S rRNA internal control (28S rRNA) and expressed as fgs RNA of a target gene per 1 ng rRNA28S. Depicted are results obtained with matched Th17 versus Th1 subsets isolated from n = 4 different HIV-uninfected individuals. Paired *t t*est values are indicated on the *graphs*. Fold change (FC) expression values in Th17 versus Th1 are included in the *graphs*

Real-time RT-PCR quantifications were performed for transcripts previously reported to be involved in the regulation of HIV replication (KLF2, PPARG), Th17 polarization (ARNTL/BMAL1), and TCR signaling (Lck, ZAP-70) as well as PTPN13, which may represent a new Th17 functional marker. Consistent with the microarrays, results in Fig. 4 demonstrate preferential expression of KLF2, PPARG, ARNTL, Lck, ZAP-70, and PTPN13 in Th17 versus Th1.

In conclusion, genome-wide transcriptional profiling revealed differences in gene expression between human Th17 and Th1 subsets, validated previously described cell-specific transcripts, and identified a large panel of new transcripts that may contribute to Th17 when compared to Th1 lineage differentiation fate and/or HIV permissiveness at post-entry levels.

### Th17 versus Th1 express higher Lck and ZAP-70 levels

**Fig. 5:**
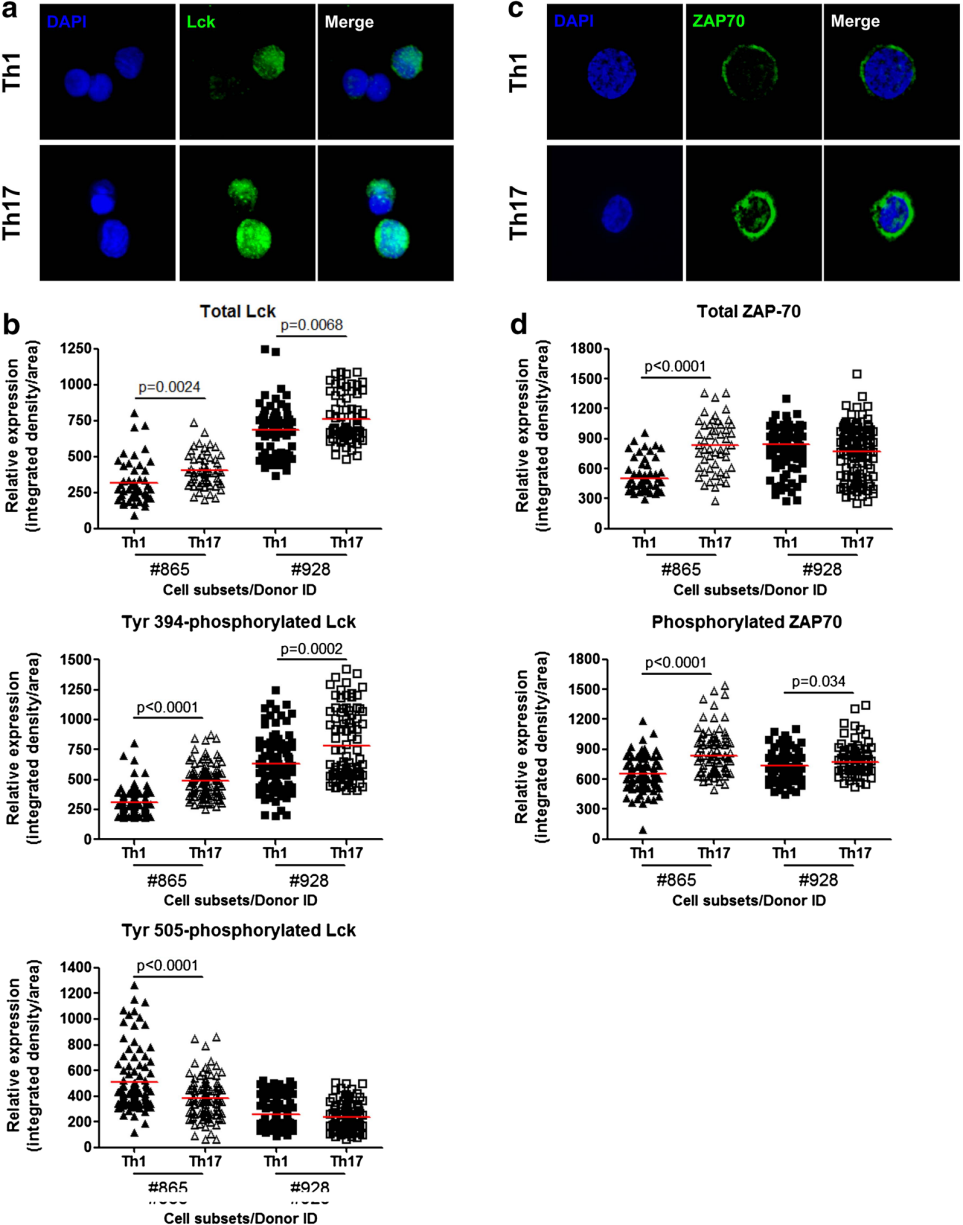
Expression of total and phosphorylated Lck and ZAP70 proteins in CCR4^+^CCR6^+^ Th17 versus CXCR3^+^CCR6^−^ Th1 cells. Matched Th17 and Th1 subsets were isolated and stimulated by CD3/CD28 Abs for 3 days as described in Fig. 1. Cells were fixed on poly-l-lysine coated slides. Intracellular staining was performed with rabbit anti-human Abs against total or phosphorylated Lck **(a, b)** and ZAP70 **(c, d)**. DAPI was used to stain cell nuclei. Slides were observed by fluorescence microscopy using a spinning-disc zeiss cell observer microscope. The visualization of total Lck (**a**) and ZAP70 (**c)** expression was performed using a 100× oil objective (NA = 1.46) in a spinning-disc confocal mode. Shown are maximum intensity z projection of z stack of matched Th17 and Th1 cells from one donor representative of observations made with cells from two different donors. The relative expression of total and phosphorylated Lck **(b)** and ZAP70 **(d)** was further quantified using the Image J software after observations made by epifluorescence with a 40× oil objective (NA = 1.40). **b**, **d** Shown are expression of total-Lck and total-ZAP70 (*upper panels*) together with phosphosphorylated Lck (Tyr 394, Tyr 505) and ZAP70 (*middle and lower panels*) in matched Th17 versus Th1 cells from two different donors (n = 50–100 cells per subsets). *Horizontal red lines* indicate median values. Unpaired *t t*est *p* values are indicated on the figures

HIV replication is restricted in resting CD4^+^ T-cells [88–90] through mechanisms that are counteracted upon activation by the TCR and/or cytokines [60, 91, 92, 93, 94, 95]. Ingenuity Pathway Analysis (IPA) performed on differentially expressed genes (p < 0.05; FC cut-off 1.3) was used to illustrate transcripts enriched in Th17 versus Th1 that are linked to TCR signaling (Additional file 7: Figure S4). The later transcripts included Lck and ZAP-70, two kinases associated with CD4 and CD3zeta, respectively, that play critical roles in the TCR downstream signaling cascade [67, 70]. ZAP-70 was reported to be essential for HIV-1 cell-to-cell transmission [71] and HIV-Nef-mediated effects [96]. The Lck and ZAP-70 mRNA levels were confirmed by RT-PCR as being significantly higher in Th17 versus Th1 (Fig. 4). Confocal fluorescence microscopy was used to visualize and quantify expression of total and phosphorylated Lck and ZAP-70 proteins in cells upon 3 days of TCR triggering. The Z-stack reconstruction of 100× images visualized the localization of Lck both extra and intra-nuclear (Fig. 5a). The expression of total Lck, together with Lck phosphorylated on Tyr394 (a positive regulatory site [97]), was significantly higher in Th17 versus Th1 from 2/2 donors (Fig. 5b, upper and middle panels). In contrast, levels of the Lck phosphorylated on Tyr505 (a negative regulatory site [97]), were significantly lower in Th17 versus Th1 from 1/2 donors (Fig. 5b, lower panel). In contrast, the localization of ZAP-70 was, as expected, mainly cytoplasmic (Fig. 5c). Although the expression of total ZAP-70 was significantly higher in Th17 versus Th1 from 1/2 donors, levels of phosphorylated ZAP-70 were significantly higher in Th17 versus Th1 from both donors (Fig. 5d). These results suggest a potential superior expression and activation status of Lck and ZAP-70 in response to TCR triggering in Th17 compared to Th1 cells. These differences are, however, minor, and this is consistent with minor differences observed in terms of Lck and ZAP-70 mRNA expression (Fig. 4).

Other downstream TCR signaling molecules overexpressed in Th17 versus Th1 include PAK2, PI3 K, and Fyn (Additional file 7: Figure S4). PAK2 is a well-established target of HIV-Nef that contributes to viral replication [65]. PI3 K is required for HIV-1 Nef-mediated down-regulation of cell surface MHC-I molecules [98]. Fyn has been demonstrated to be involved in NF-κB mediated HIV transcription [99]. In contrast, Grb2 (growth factor receptor-bound protein 2) was found down regulated in Th17 versus Th1 (Additional file 7: Figure S4). This is consistent with the fact that Grb2 inhibits the Tat-mediated transactivation of HIV-1 LTR and subsequent viral replication [100] (Additional file 4: Table S3).

Altogether, these results demonstrate superior expression of signaling molecules associated with TCR signaling in Th17 versus Th1, including the active phosphorylated forms of Lck and ZAP-70. These differences very likely contribute to the superior ability of Th17 when compared to Th1 to respond to weak TCR signals, thus creating a cellular environment favorable to HIV replication.

### Th17 versus Th1 exhibit superior NF-κB nuclear translocation and DNA-binding activity

**Fig. 6:**
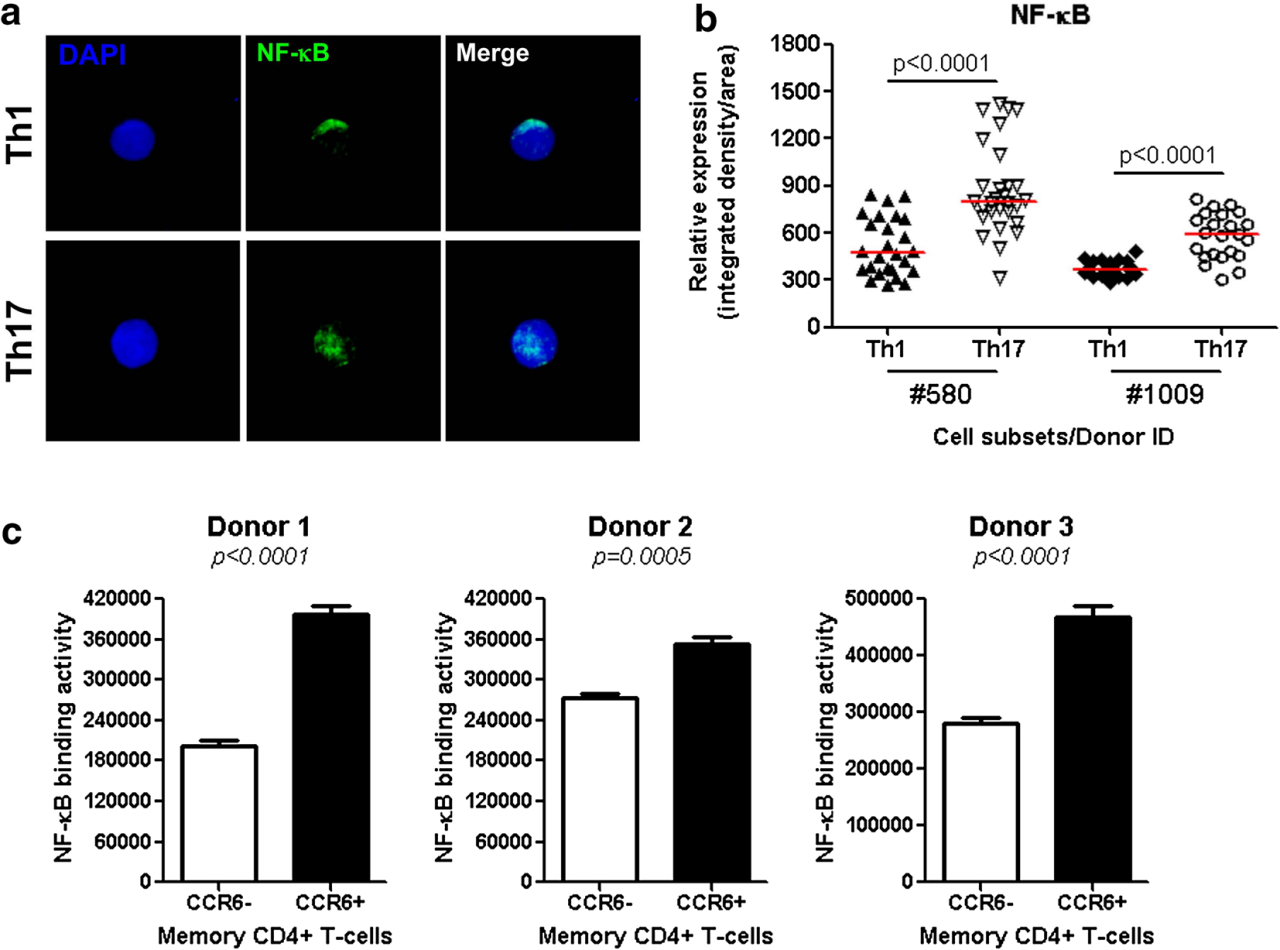
NF-κB nuclear translocation and DNA-binding activity in CCR4^+^CCR6^+^ Th17 versus CXCR3^+^CCR6^−^ Th1 cells. **a**, **b** Matched Th17 and Th1 subsets were isolated and stimulated via CD3/CD28 for 3 days as described in Fig. 1. Cells were seeded on poly-l-lysine coated slides, and intracellular staining was performed with rabbit anti-human NF-kB followed by goat anti-rabbit AlexaFluor 488 Abs. Slides were mounted with the *ProLong Gold Antifade* reagent containing the nuclear dye DAPI. Slides were then observed by confocal microscopy. **a** NF-κB expression was observed under a 100× oil-immersion objective (NA = 1.46) in a spinning-disc confocal mode system. Shown are maximum intensity z projection of z stack of Th17 and Th1 cells from one donor representative of observations made with two different donors. **b** The relative expression of intra-nuclear NF-κB was quantified in cells from two different donors using the Image J software based on observations by epifluorescence with a 40x Oil immersion objective (NA = 1.40). Horizontal red lines indicate median values. Unpaired *t t*est *p* values are indicated in the figure. **c** Memory CCR6^+^ and CCR6^−^ CD4^+^ T-cell subsets were enriched by MACS and sorted by FACS as previously reported [104]. Cells were stimulated via CD3/CD28, as in Fig. 1, and nuclear fractions were extracted. Shown is the ELISA quantification of NF-κB-p65 DNA-binding activity in nuclear extracts from matched CCR6^+^ and CCR6^−^ subsets isolated from three different HIV-uninfected donors (mean ± SD of triplicate wells). Student *t t*est *p* values are indicated in the figures

The nuclear translocation of the transcription factor NF-κB is a key event in the signaling cascade of downstream TCR. NF-κB regulates transcription of numerous genes involved in T-cell activation and survival [101], and also binds to the HIV promoter to initiate viral genome transcription [102, 103]. Of note, transcripts for mucosa associated lymphoid tissue lymphoma translocation gene 1 (MALT1), a paracaspase critical for NF-κB activation [68, 69], were enriched in Th17 versus Th1 (Additional file 1: Table S1). This evidence, together with the up-regulation of TRIM8 [82] transcripts in Th17 versus Th1, suggests superior NF-κB activity in Th17 cells. To investigate this possibility, we first used confocal fluorescence microscopy to visualize and quantify expression of NF-κB in the nuclei of Th17 versus Th1 upon TCR triggering. In both Th17 and Th1, NF-κB was mainly localized in the nucleus; however, the intensity of intra-nuclear staining was significantly higher in Th17 than Th1 in 2/2 donors (Fig. 6a, b). In parallel, an ELISA-based assay was used to quantify the NF-κB DNA-binding activity in the nuclear fractions isolated from TCR-activated memory CD4^+^ T-cells expressing or lacking the Th17 marker CCR6 (Fig. 6c). Of note, the CCR6^−^ fraction includes Th1 cells, while the CCR6^+^ fraction includes Th17 cells. The purity of nuclear fractions was assessed by western blotting; this demonstrated the expression of the nuclear marker Histone H3 but the absence of the cytoplasmic marker β-actin (data not shown). For equal quantities of total nuclear proteins, the NF-κB DNA-binding activity was significantly higher in CCR6^+^ than CCR6^−^ T-cells in 3/3 subjects (Fig. 6c). These results are consistent with previous findings by our group that CCR6^+^ compared to CCR6^−^ T-cells are major HIV replication targets [104]. Thus, TCR triggering results in superior NF-κB nuclear translocation and DNA-binding activity in Th17 when compared to Th1. This difference highly likely contributes to enhanced transcription of the HIV genome in Th17 versus Th1.

### Th17 versus Th1 proliferate in response to low intensity TCR triggering

**Fig. 7:**
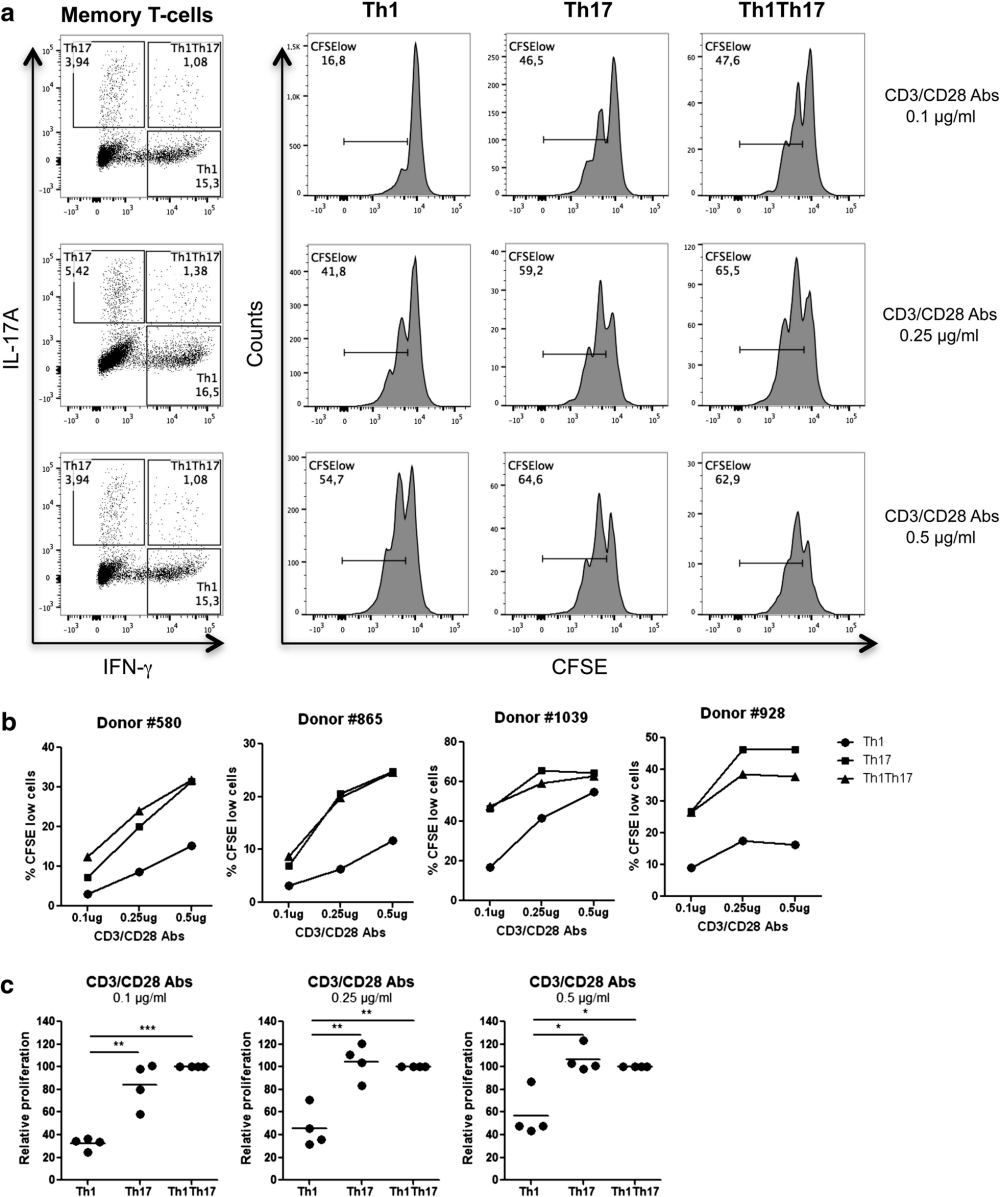
Proliferation of CD4^+^ T-cells expressing IL-17A versus IFN-γ in response to low TCR triggering. Memory CD4^+^ T-cells were isolated from PBMCs of four different HIV-uninfected donors by negative selection using magnetic beads. Cells were loaded with CFSE (0.5 µM) and cultured in the presence of different doses of immobilized CD3 and soluble CD28 Abs (0.1, 0.25, or 0.5 µg/ml) for 3 days. Cells were then stimulated with PMA/Ionomycin in the presence of Brefeldin A for 18 h. Intracellular staining was performed with IL-17A and IFN-γ Abs. Based on the expression of IL-17A and/or IFN-γ, three cell subsets were identified as follows: Th1 (IL-17A^−^IFN-γ^+^), Th17 (IL-17A^+^IFN-γ^−^), and Th1Th17 (IL-17A^+^IFN-γ^+^). The frequency of proliferating cells (CFSE^low^) was analyzed in each of the three cytokine-expressing subsets. **a** Shown are flow cytometry results in one representative donor out of four. Shown are statistical analyses of absolute **(b)** and relative (Th1Th17 proliferation levels were considered 100 %) **c** proliferation (CFSE^low^) levels in Th1, Th17, and Th1Th17 cells from four different donors. Paired *t t*est p values are indicated on the *graphs*

The engagement of TCR induces complex modifications of cellular processes, including the production of effector cytokines and ultimately cell proliferation. In an effort to determine whether superior levels of Lck/ZAP-70 phosphorylation (Fig. 5) and NF-κB activation (Fig. 6) impact on the proliferation potential of Th17 versus Th1 cells, a CFSE dilution assay was used to measure at the single-cell level the proliferation of memory CD4^+^ T-cells with intracellular expression of IL-17A only (Th17), IFN-γ only (Th1), or both IL-17 and IFN-γ (Th1Th17). Optimal IL-17A expression was observed when cells were stimulated with CD3/CD28 Abs for three days and with PMA and Ionomycin in the presence of Brefledin A for additional 16 h (Fig. 7a). Th17 and Th1Th17 versus Th1 proliferated at superior levels in 4/4 donors, and this was for three different concentrations of CD3 and CD28 Abs (0.1, 0.25, and 0.5 µg/ml); differences were more remarkable for the lowest CD3/CD28 Abs concentration (Fig. 7a–c). Therefore, our results provide evidence that Th17 versus Th1 exhibit a superior ability to proliferate and produce IL-17A in response to relatively low TCR triggering signals. Our results are consistent with previous studies by other groups reporting on the induction of Th17 effector functions upon low strength TCR activation [105, 106].

### MAP3K4, PTPN13, and SERPINB6 act as HIV permissiveness factors

**Fig. 8:**
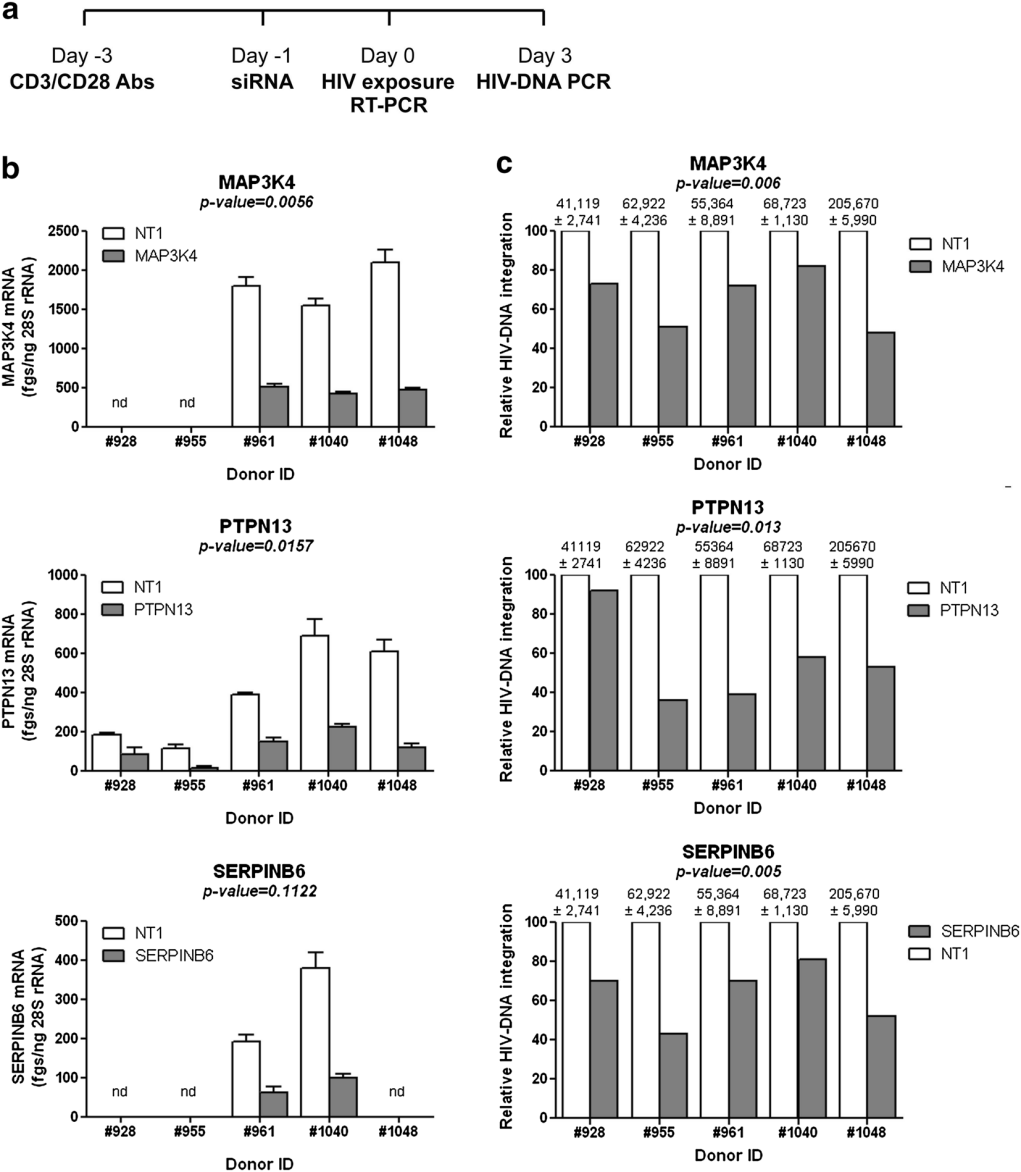
Effects of MAP3K4, PTPN13, and SERPINB6 RNA interference on HIV-1 integration in memory CD4^+^ T cells. RNA interference experiments were performed on memory CD4^+^ T-cells as described in the Fig. 7 legend. **a** Shown is the experimental flow chart. Briefly, cells were stimulated via CD3/CD28 for 2 days, washed and nucleofected with siRNA pools (1 µM) specific for MAP3K4, PTPN13, and SERPINB6 or a non-targeting siRNA (NT). Nucleofected cells were cultured in the presence of IL-2 (5 ng/ml) for 24 h and then exposed to the HIV NL4.3BAL-GFP strain (50 ng HIV-p24/ml) for 3 h at 37 °C. Infected cells were cultured in the presence of IL-2 (5 ng/ml) for 3 days. **b** The yield of siRNA silencing was determined by real-time RT-PCR quantification of MAP3K4, PTPN13, and SERPINB6 mRNA expression in cells nucleofected with targeting (MAP3K4, PTPN13, SERPINB6) versus non-targeting (NT) siRNA. Shown are results obtained with cells from two to five different donors. **c** Levels of integrated HIV-DNA were quantified by nested real-time PCR in cells harvested at day three post-infection. HIV-DNA copy numbers were normalized relative to CD3 expression. Shown is relative HIV-DNA integration in targeting versus NT siRNA conditions (normalized to the maximal value considered to be 100 % in NT condition); the values above graphs are integrated HIV-DNA copies per 10^6^ cells in NT nucleofected cells (mean ± SD of triplicate wells). Paired *t t*est values are indicated on the *graphs*

Other differentially expressed transcripts included MAP3K4 and PTPN13 that were up regulated (Additional file 5: Figure S2) and SERPINB6 that was down regulated in Th17 versus Th1 (Tables 1, 2). Of note, SERPINB6, an intracellular serine protease inhibitor, was previously identified as an HDF in genome-wide siRNA screens performed in HeLa cells [58]. We assessed the effect of RNA interference against MAP3K4, PTPN13, and SERPINB6 on the efficacy of HIV-DNA integration in memory CD4^+^ T-cells. The results in Fig. 8 demonstrate that decreased expression of MAP3K4, PTPN13, and SERPINB6 mRNA was associated with a statistically significant reduction in levels of integrated HIV-DNA; this indicates that these molecules restrict viral replication prior to integration. Thus, in addition to the identification of MAP3K4 and PTPN13 as novel positive regulators of HIV replication preferentially expressed in Th17 cells, we confirm SERPINB6 is indeed a HDF. The detailed molecular mechanisms by which MAP3K4 and PTPN13 regulate HIV integration in Th17 cells remain to be investigated.

## Discussion

HIV-1 targets for infection and subsequent depletion cells of the immune system that play key roles in the defense against pathogens, including the Th17-polarized CD4^+^ T-cells [19, 37, 38, 50]. The unique developmental plasticity (e.g., ability to convert into Th1, Th2 and Tregs [107, 108]), pathogen-specificity and functional heterogeneity [13, 14, 15, 109, 110, 111, 112], together with their long-lived properties [52–54], position Th17 cells at the very core of the immune system. Given their predominant location at mucosal surfaces, including the gut-associated lymphoid tissues (GALT), Th17 cells represent the first HIV/SIV targets at the portal sites of entry [25, 113]. Quantitative and qualitative alterations in Th17 cells within the GALT represent a major cause of HIV/SIV disease progression [24, 25, 114, 115, 116, 117, 118, 119]. Therefore, understanding molecular mechanisms of HIV permissiveness in Th17 cells represents a major research priority. Studies by our group [37], confirmed by others [38, 50], demonstrated that CCR4^+^CCR6^+^Th17 and CXCR3^+^CCR6^+^Th1Th17 cells are highly permissive, while CXCR3^+^CCR6^−^Th1 are relatively resistant to R5 and X4 HIV infection, and CCR4^+^CCR6^−^Th2 are permissive to X4 HIV only [37]. In this manuscript, we used a systems biology approach to unveil molecular mechanisms of HIV replication in Th17 cells by comparing their transcriptome to those of Th1, Th2 and Th1Th17 cells. We reveal here for the first time to our knowledge a molecular signature associated with HIV permissiveness in primary Th17 cells.

Our genome-wide transcriptional profiles demonstrated superior expression of KLF2 in Th17 compared to Th1 cells. These findings were confirmed by RT-PCR. KLF2 is a transcription factor that binds to the CCR5 promoter and positively regulates its expression and the subsequent permissiveness of CD4^+^ T-cells to R5 HIV [76]. CCR5 is indeed a key co-receptor for HIV entry [120] and one of the major determinants of disease progression [121, 122] involved in the early phases of HIV acquisition at mucosal surfaces [123, 124]. Activated CCR5^+^ T-cells are enriched within the GALT and represent the first HIV targets [125]. However, superior HIV permissiveness in Th17 versus Th1 is not the reflection of their superior CCR5 expression ex vivo [37]; this suggests that CCR5 is essential but not sufficient to support R5 HIV entry and/or subsequent replication. Nevertheless, the stability of CCR5 expression upon TCR triggering in long-term cultures was not investigated in our system, and therefore the potential role of KLF2 in regulating superior/stable CCR5 expression in Th17 cells cannot be excluded. The autocrine production of CCR5 ligands was previously identified as a mechanism by which CMV-specific CD4^+^ T-cells are protected from HIV infection [126]. The overexpression of transcripts for CCR5 ligands observed in this study in Th1 versus Th17 is consistent with previous reports by our group [37] and others [50, 114] where CCR5 ligand protein levels were investigated. Thus, the autocrine production of CCR5 ligands by Th1 cells may contribute to limited HIV entry in Th1 cells. Despite any potential regulatory mechanisms at entry level, we report superior HIV-DNA integration in Th17 versus Th1 cells upon exposure to both wild-type and VSV-G-pseudotyped HIV; this indicates that post-entry mechanisms contribute to viral permissiveness in Th17 cells.

Upon receptor-mediated entry, HIV-1 uses the host-cell molecular machinery to ensure its reverse transcription, integration and transcription [59, 60]. Large siRNA screens performed on cell lines identified networks of HDFs acting at different levels of the viral life cycle [56, 57, 58, 84]. Very few HDF identification studies were performed on primary cells [127–129]. JAK1 is one of the very few HDFs identified in more than two siRNA screens that is also included in the NCBI HIV interaction database [60]. Of particular importance, our microarrays revealed an up-regulation of JAK1 transcripts in Th17 versus Th1. JAK1 is a tyrosine kinase associated with the signaling through the receptors of type I and II cytokines; activation of JAK1 induces STAT3 phosphorylation in response to IL-21 stimulation [130]. Consistent with JAK1 up-regulation in Th17 cells, the JAK signaling pathway is altered in subjects with Hyper IgE syndrome that exhibit mutations in STAT3 and subsequent Th17 deficiency [131]. JAK1 antagonists were reported to interfere with Th17 polarization in a mouse model of psoriasis [132]. Considering the fact that JAK antagonists inhibit HIV replication and reactivation [133], JAK1 may represent a novel therapeutic target to interfere with infection in Th17 cells.

Despite a low degree of overlap among individual HDFs identified in different siRNA screens [56, 57, 58, 84], pathways such as NF-κB, peroxisome proliferator-activated receptor (PPAR), and retinoic acid receptor were identified as being important for HIV permissiveness in at least two distinct studies [59, 60]. A previous study by our group demonstrated that the transcription factor PPARγ is expressed at superior levels in Th1Th17 versus Th1 and acts as a negative regulator of HIV replication [129]. Of note, PPARγ is also an intrinsic negative regulator of Th17 polarization [134]. Therefore, mechanisms involved in Th17 polarization and HIV replication are overlapping. The present transcriptional profiles consistently demonstrated superior expression of PPARγ in Th17 versus Th1 cells. These findings provide further evidence that Th17, similar to Th1Th17 cells [129], are endowed with intrinsic mechanisms that control HIV permissiveness, mechanisms that should be targeted therapeutically.

Consistent with differential HIV replication in Th17, Th1Th17, Th1, and Th2 memory CD4^+^ T-cell subsets [37], the present genome-wide transcriptional profiling revealed the remarkable transcriptional differences between Th17 and Th1 cells. Gene set variation analysis revealed unique pathways enriched in Th17 versus Th1 cells, including TCR signaling, T-helper differentiation, IL-7 signaling, nuclear receptor transcription, and circadian repression of expression by REV-ERBα. Therefore, it is reasonable to assume that pathways preferentially expressed in Th17 cells are exploited by HIV for successful replication.

Similar to Th2 and in contrast to Th1 [135], Th17 polarization depends on low strength TCR signals [105]. Consistently, we found an enriched expression of transcripts linked to the TCR signaling cascade in Th17 versus Th1, including the major kinases Lck and ZAP-70. These differences were validated by RT-PCR at the population level and further visualized/quantified at the single-cell level by confocal microscopy. Of note, Lck facilitates assembly of HIV-1 by targeting HIV-1 Gag to the plasma membrane in T cells [136], while ZAP-70 kinase regulates HIV cell-to-cell spread and virological synapse formation [71]. Superior expression of phosphorylated active forms of Lck and ZAP-70 in Th17 versus Th1 cells coincided with superior expression of transcripts for multiple kinases and phosphatases downstream from the TCR. Among transcripts associated with the TCR reactome, our RNA interference experiments identified MAP3K4 and PTPN13 as positive regulators of HIV replication in Th17 cells. The phosphorylation of p38 MAPK by MAP3K4 was previously linked to Th17 polarization signals [137]. We previously reported MAP3K4 up-regulation in HIV-permissive Th1Th17 cells [129]. PTPN13, a tyrosine phosphatase involved in the negative regulation of Fas-dependent apoptosis upon TCR triggering [138–140], was identified as a Th1Th17 marker [62, 129]. Here, we confirmed by RT-PCR the exclusive expression of PTPN13 mRNA in Th17 versus Th1. Mechanisms by which MAP3K4 and PTPN13 regulate HIV replication remain to be further examined, but they are likely linked to the control of the state of cellular activation upon TCR engagement.

The replication of HIV is limited in resting CD4^+^ T-cells [91] through restriction mechanisms that are abrogated by activation/proliferation induced upon engagement of the TCR and/or cytokine receptors [141–144]. Our functional studies demonstrated that CD3/CD28 engagement resulted in superior cell proliferation and NF-κB nuclear translocation as well as DNA binding activity in Th17 versus Th1. Indeed, in contrast to IFN-γ^+^, IL-17A^+^ cells proliferated efficiently in response to low concentrations of CD3/CD28 Abs. A study by Santarlasci et al. reported an impaired ability of Th17 cells to proliferate [145]. This report is in contrast to our results on superior NF-κB activation and proliferation in Th17 versus Th1 cells. These discrepancies may be explained by results generated with polyclonal subsets producing IL-17 ex vivo in our studies versus Th17 clones in studies by Santarlasci et al. 145], clones that are potentially exhausted or senescent due to long-term maintenance in vitro. Our findings are in line with publications by other groups on the long lived properties of Th17 cells [52–54]. In addition to the ability of NF-κB to regulate transcription of multiple host genes that may be critical for HIV permissiveness, NF-κB directly binds to the HIV promoter and positively regulates its activity [102, 103]. Accordingly, genome-wide siRNA screens for HDFs identified the NF-κB pathway as being a key regulator of HIV permissiveness [59, 60]. In our transcriptional studies, MALT1 (a paracaspase involved in NF-κB activation [68]) and TRIM8 (a regulator of NF-κB and STAT3-dependent signaling cascades [81, 82] and documented HDF [57]) were also found up regulated in Th17 versus Th1 cells. Of particular interest, MALT1 was recently linked to Th17 polarization [69]. These results emphasize complex host cell-pathogen interactions by which HIV takes advantage of universal and Th17-specific proximal/distal components of the TCR signaling cascade for its efficient replication.

Our findings that Th17 cells proliferate in response to weak TCR signals are consistent with recent studies demonstrating that miR-181a, a microRNA involved in the regulation of TCR activation [146], is preferentially induced under Th17 polarizing conditions [106]. MiR-181a lowers the TCR activation threshold through the modulation of ERK phosphorylation [106]. Of note, miR-181a is also involved in the post-transcriptional regulation of SAMHD1 [147], an HIV restriction factor expressed in quiescent CD4^+^ T-cells [148] that limits HIV reverse transcription by its dNTPase [149] and RNase activity [150]. The restriction ability of SAMHD1 is negatively regulated by phosphorylation [151]. Our microarrays were not designed to detect microRNAs and did not reveal differences in SAMHD1 mRNA expression. Further studies are needed to clarify the potential role of SAMHD1 in controlling HIV replication in Th17 cells.

## Conclusions

This study reveals a unique molecular signature of HIV permissiveness in Th17 cells (e.g., PPARG, PAK2, KLF2, PTEN, ITGB7, ATG16L1, Alix/AIP1/PDCD6IP, LGALS3, JAK1, TRIM8, MALT1, FOXO3, ARNTL, ABCB1, TNFSF13B/BAFF, and CDKN1B) and provides evidence that a unique TCR signaling cascade is favorable to HIV replication in Th17 cells. The current identification of novel Th17-specific HDFs is instrumental for designing novel therapeutic strategies aimed at interfering with viral replication, while maintaining the Th17 role in mucosal immunity.

## Methods

### Subjects

Healthy HIV-uninfected donors were recruited at the Montreal Chest Institute, McGill University Health Centre, and Centre Hospitalier de l’Universite de Montreal (CHUM, Montreal, Quebec, Canada). Large quantities of PBMCs (10^9^–10^10^ cells) were collected by leukapheresis as previously described [152].

### Ethics statement

This study, using PBMC samples from healthy HIV-uninfected subjects, was conducted in compliance with the principles included in the Declaration of Helsinki. This study received approval from the Institutional Review Board of the McGill University Health Centre and the CHUM-Research Centre, Montreal, Quebec, Canada. All human subjects that donated biological samples for this study provided written informed consent for their participation in the study. All human subjects agreed with the publication of the subsequent results generated using the samples.

### Flow cytometry analysis

Fluorochrome-conjugated Abs used for polychromatic flow cytometry analysis were CD3-Pacific Blue (UCHT1), CD4-Alexa700 (RPA-T4), CD45RA-APC-Cy7 (custom), CCR4-PE-Cy7 (1G1), CXCR3-PE-Cy5 (1C6), CCR6-PE (11A9), Ki67-FITC, IFN-γ-AlexaFluor 700 (B27) (BD Pharmingen), CD56-FITC (MEM188), IL-17-PE (64DEC17) (eBioscience), HIV-p24-FITC (FH190-1-1) (Beckman Coulter), CD8-FITC (BW135/80), and CD19-FITC (LT19) (Miltenyi). A viability dye (Molecular Probes^®^ LIVE/DEAD^®^ Fixable Dead Cell Stain Kits, Invitrogen) was used to exclude dead cells. Cells were stained and analyzed by FACS using the BD LSRII cytometer and the FlowJo software, as previously described [129].

### Magnetic (MACS) and fluorescence activated cell sorting (FACS)

Total or memory CD4^+^ T-cells were enriched from PBMC by negative selection using magnetic beads (MACS, Miltenyi), with a purity >95 % as previously described [37, 104]. Then, cells were stained with CD45RA-APC-Cy7, CCR6-PE, CCR4-PE-Cy7, CXCR3-PE-Cy5 Abs and a cocktail of FITC-conjugated Abs to exclude CD8^+^ T-cells (CD8), NK cells (CD56), and B cells (CD19). The sorting gates were set on FITC^neg^ memory (CD45RA^neg^) T-cells. Four subsets were sorted by flow cytometry (BDAria II): CXCR3^+^CCR4^−^CCR6^−^ (CXCR3^+^Th1), CXCR3^−^CCR4^+^CCR6^−^ (CCR4^+^Th2), CXCR3^−^CCR4^+^CCR6^+^ (CCR4^+^CCR6^+^Th17), and CXCR3^+^CCR4^−^CCR6^+^ (CXCR3^+^CCR6^+^Th1Th17). In other experiments, memory CCR6^+^ and CCR6^−^ T-cells were sorted upon staining with CD45RA-APC-Cy7 and CCR6-PE Abs as well as a mixture of FITC-conjugated CD8, CD56, and CD19 Abs. A viability dye was used to exclude dead cells. Post-sort FACS analysis demonstrated sorted T-cell subsets were >99 % pure, as reported earlier [37, 104, 129].

### Genome-wide transcriptional profiling

Matched memory CD4^+^ T-cell subsets were isolated by FACS from five different HIV-uninfected donors and stimulated with immobilized CD3 and soluble CD28 (1 µg/ml) for 3 days. Total RNA was isolated using RNeasy columns kit (Qiagen) according to the manufacturer’s protocol. RNA quantity was determined by Pearl nanophotometer (Implen, Germany) (10^6^ cells yielded 1–5 µg RNA). Genome-wide analysis of gene expression was performed on total RNA by Génome Québec (Montreal, Quebec, Canada). Briefly, the quality of total RNA was tested using the Agilent 2100 Bioanalyzer chip. High quality RNA was reverse transcribed and hybridized on the Illumina HumanHT-12 v4 Expression BeadChip providing coverage for more than 47,000 transcripts and known splice variants across the human transcriptome.

### Transcriptional profiling analysis

Gene expression analyses were performed as previously described [129]. Briefly, after quality control of the microarray data, the resulting expression matrix was used as input for linear modelling using Bioconductor’s limma package, which estimates the fold-change among predefined groups by fitting a linear model and using an empirical Bayes method to moderate standard errors of the estimated log-fold changes for expression values from each gene. A linear mixed model was designed with the population as a fixed effect and the donor ID as a random effect. P values from the resulting comparison were adjusted for multiple testing according to the method of Benjamini and Hochberg (1995). This method controls the false discovery rate (FDR), which was set to 0.05 in this analysis. Determination of regulated gene expression is based on p values or adjusted p values as indicated in the figure or table legends. The entire microarray dataset and technical information requested by Minimum Information About a Microarray Experiment (MIAME) are available at the Gene Expression Omnibus (GEO) database under accession number GSE70396. Differentially expressed genes (cut-off 1.3-fold; p < 0.05) were classified through Gene Ontology using the NetAffx web-based application (Affymetrix). Corresponding heat maps for biological function categories were generated using programming language R. Enrichment Statistics (ES) from the gene set variation analysis were calculated as the maximum distance of the random walk statistic using the GSVA bioconductor package [153] on the same databases described in Bernier et al. [129]. Differential expression analysis of the ES was performed with the limma bioconductor package following the same model applied to probe-level expression. The gene networks were generated through the use of ingenuity pathways analysis (Ingenuity^®^ systems, http://www.ingenuity.com).

### Real-time RT-PCR

One step SYBR Green real-time RT-PCR (Qiagen) was carried out in a LightCycler 480 II (Roche) according to manufacturer’s recommendations, as we previously reported [37, 104, 129]. Briefly, for standard curve preparation, 5-50 ng of total RNA were reverse transcribed using a SYBR Green mix (Qiagen) containing 0.5 μM primers. Agarose gel electrophoresis was used to visualize the size of the amplification products. cDNA purification was performed using the QIAquick Gel Extraction Kit (Qiagen). Serial dilutions of cDNA (20,000; 2000; 200; 20; 2; 0.2 fgs) were used for the absolute quantification of target gene expression. QuantiTect Primer Assays for KLF2, PPARγ, ARNTL, ZAP-70, Lck, PTPN13, RORC, MAP3K4, and SERPINB6 were purchased from Qiagen. The expression of each gene was normalized relative to the internal control 28S rRNA levels (forward 5′-CGAGATTCCTGTCCCCACTA-3′; reverse 5′-GGGGCCACCTCCTTATTCTA-3′, IDT). Melting curve analysis performed after real-time amplification revealed the uniformity of thermal dissociation profile for each amplification product. Samples without template or without reverse transcriptase were used as negative controls. Each RT-PCR reaction was performed in triplicate.

### HIV infection and quantification of viral replication

The following HIV-1 molecular clones were used in this study: (1) replication-competent CCR5-using (R5) HIV NL4.3BAL; (2) replication-competent R5 NL4.3BAL-GFP expressing *gfp* in place of *nef*; and (3) single-round VSVG-HIV-GFP, an *env*-deficient NL4.3 provirus pseudotyped with the VSV-G envelope and expressing *gfp* in place of *nef* [37, 104, 129]. HIV stocks were produced, titrated, and used to infect cells (50 ng HIV-p24 per 10^6^ cells) as previously described [37, 104, 129]. HIV-p24 levels were quantified in cell culture supernatants using a homemade ELISA [37, 104]. HIV-DNA integration was quantified in cell lysates by real-time nested PCR (10^5^ cells per test in triplicate; detection limit: three HIV-DNA copies), as previously described [37, 104, 129, 154].

#### Fluorescence microscopy and quantitative image analysis

The visualization and quantification of protein expression was performed by confocal microscopy, as previously described [129]. Briefly, FACS-sorted Th17 and Th1 subsets were stimulated via CD3/CD28 for 3 days (1 μg/ml) and placed into poly-l-lysine-coated eight-wells glass culture slides (BD Biosciences) (10^5^ cells/well). Cells were stained with primary Abs against total Lck (clone 73A5), total ZAP-70 (clone 99F2), phosphorylated Lck on Tyr-394 (Santa Cruz Biotechnology) and Tyr-505 (clone 2751), phosphorylated ZAP-70 on Tyr319 (clone 65EA cross reacting with phosphorylated Syk on Tyr352), and NF-κB p65 (clone 3034) and Alexa Fluor 488-conjugated goat anti-rabbit Abs (Invitrogen) as secondary Abs. The above Abs were purchased from Cell Signaling Technology, unless otherwise specified. Slides were mounted using ProLong Gold Antifade medium with the nuclear dye DAPI (Invitrogen, Molecular Probes). Epi-fluorescent and Spinning Disc confocal microscopy images were acquired out on an automated Cell Observer Z1^®^ microscope (Carl Zeiss) using the AxioVision 4.8.2 software (Carl Zeiss). For the analysis of protein cellular localization, spinning disc confocal images were acquired using the 100× oil immersion objective (numerical aperture, NA: 1.46) and maximum intensity projection of 0.2 μm z-stack sections were realized using ImageJ software (NIH) after background subtraction. For statistical analysis of protein expression, random epi-fluorescent images were acquired with the 40× oil immersion objective (NA: 1.3). All acquisitions between the different T-cell subsets were performed with the same illumination status in the same run. Integrated density was measured after background subtraction with ImageJ software. Data were compared by analysis of integrated density/area for 50–100 cells/subset.

#### NF-κB DNA-binding activity

Nuclear extracts were obtained from activated CD4^+^ T-cells using the BD transfactor extraction kit (Clonetech Laboratories). The active form of NF-κB p65 was quantified by ELISA (1 μg nuclear protein/test; Assay Designs & Stressgen). The specificity of NF-κB p65 DNA-binding was determined using wild-type and mutated NF-κB p65 duplex competitors, according to the manufacturer’s protocol.

#### CFSE dilution assay and intracellular cytokine staining

Cell proliferation was measured using the Carboxy Fluorescein Succinimidyl Ester (CFSE) dilution assay, as previously described [155]. Briefly, memory CD4^+^ T-cells were loaded with CFSE and cultured in the presence of different doses of immobilized CD3 and soluble CD28 Abs (0.5, 0.25, and 0.1 µg/ml) for 1, 2, 3, or 4 days. Cells were further stimulated with PMA (50 ng/ml, Sigma) and Ionomycin (1 µg/ml, Sigma) in the presence of Brefeldin A (2 μg/ml, Sigma) for 18 h. The production of IL-17A and IFN-γ was measured by intracellular staining with appropriate Abs using the BD cytofix/cytoperm fixation/permeabilization solution kit (BD Biosciences) according to the manufacturer’s protocols.

#### RNA interference

RNA interference studies were performed as described earlier [129]. Briefly, PBMCs were thawed and rested overnight at 37 °C. Memory CD4^+^ T-cells were isolated from PBMC by negative selection using magnetic beads (Miltenyi Biotec). Cells were stimulated by CD3/CD28 Abs for 2 days and nuclofected with 100 µM specific (MAP3K4, PTPN13, SERPINB6) or non-targeting (NT1) siRNA (ON-TARGETplus SMART pool, Dharmacon) using the Amaxa Human T cell Nucleofector Kit (Amaxa, Lonza), according to the manufacturer’s protocol. Cells were suspended in the NF solution (100 µl/2 × 10^6^ cells) and nucleofected using the Amaxa Nucleofector II Device and the human activated T-cell protocol (T-20). Cells (2 × 10^6^) were transferred into 48-well plates containing 1 ml of RPMI1640 (10 % FBS, 5 ng/ml IL-2, w/o antibiotics) and cultured for 24 h at 37 °C. Cells were exposed to HIV and cultured up to 9 days. Culture supernatants were harvested and media was refreshed every 3 days. The effectiveness of RNA silencing was assessed by SYBR Green real-time RT-PCR 24 h post-nucleofection. IL-17A production in cell supernatants was measured by ELISA at day 3 post-infection. Five days post-nucleofection, cells were stained with LIVE/DEAD^®^ Fixable Dead Cell Stain Kit (invitrogen) and intracellular staining was performed using Ki67 Abs. Cell viability (vivid-) and cell cycle progression (Ki67^+^) were analyzed by FACS (BD LSRII).

### Statistics

All statistical analyses were performed using the Prism 5 (GraphPad software). Specifications are included in the figure legends.

## Accession numbers

The entire microarray dataset and technical information requested by Minimum Information About a Microarray Experiment (MIAME) are available at the Gene Expression Omnibus (GEO) database under accession number GSE70396.

## Additional files

10.1186/s12977-015-0226-9 The complete list of genes up regulated in Th17 versus Th1 subsets are included and classified based on p-values < 0.05 and FC cut-off 1.3.
10.1186/s12977-015-0226-9 The complete list of genes down regulated in Th17 versus Th1 subsets are included and classified based on p-values < 0.05 and FC cut-off 1.3.
10.1186/s12977-015-0226-9 **a** A heat map including the hierarchical clustering of all differentially expressed genes in Th17 versus Th1 subsets and **b** a heat map including top differentially expressed canonical pathways in Th17 versus Th1 subsets identified using Gene set variation analysis (GSVA; adj. p-values < 0.05).
10.1186/s12977-015-0226-9 The complete list of canonical pathways differentially expressed in Th17 versus Th1 subsets were identified using GSVA and classified based on adj. p-values < 0.05 and log FC.
10.1186/s12977-015-0226-9 Included are top canonical pathways (C2) (a), transcription factors (C3) (b), and biological processes (C5) (c) that are differentially expressed between Th17 and Th1 subsets and were identified using Gene set enrichment analysis (GSEA).
10.1186/s12977-015-0226-9 A meta-analysis for transcripts included in the NCBI HIV Interaction data base that were identified as top differentially expressed between Th17 and Th1 subsets.
10.1186/s12977-015-0226-9 The T cell receptor signalling pathways was generated using the Ingenuity pathway analysis (IPA), with transcripts up and down regulated in Th17 versus Th1 subsets being highlighted in red and green, respectively.
