# Supplementary figures and images for: Identification of novel HIV-1 dependency factors in primary CCR4+CCR6+Th17 cells via a genome-wide transcriptional approach
Source: Retrovirology. 2015 Dec 10;12:102. doi: 10.1186/s12977-015-0226-9 (PMC4676116; doi:10.1186/s12977-015-0226-9)

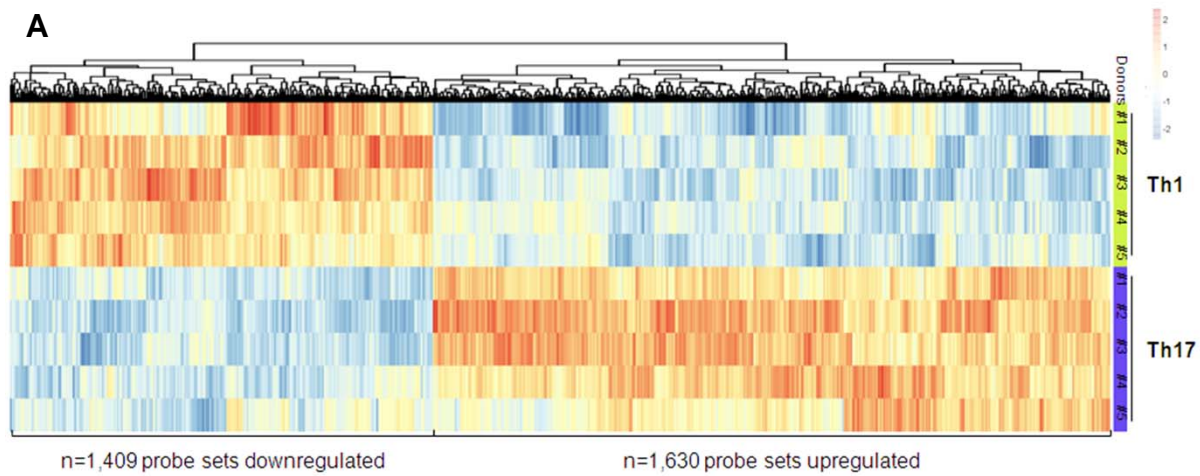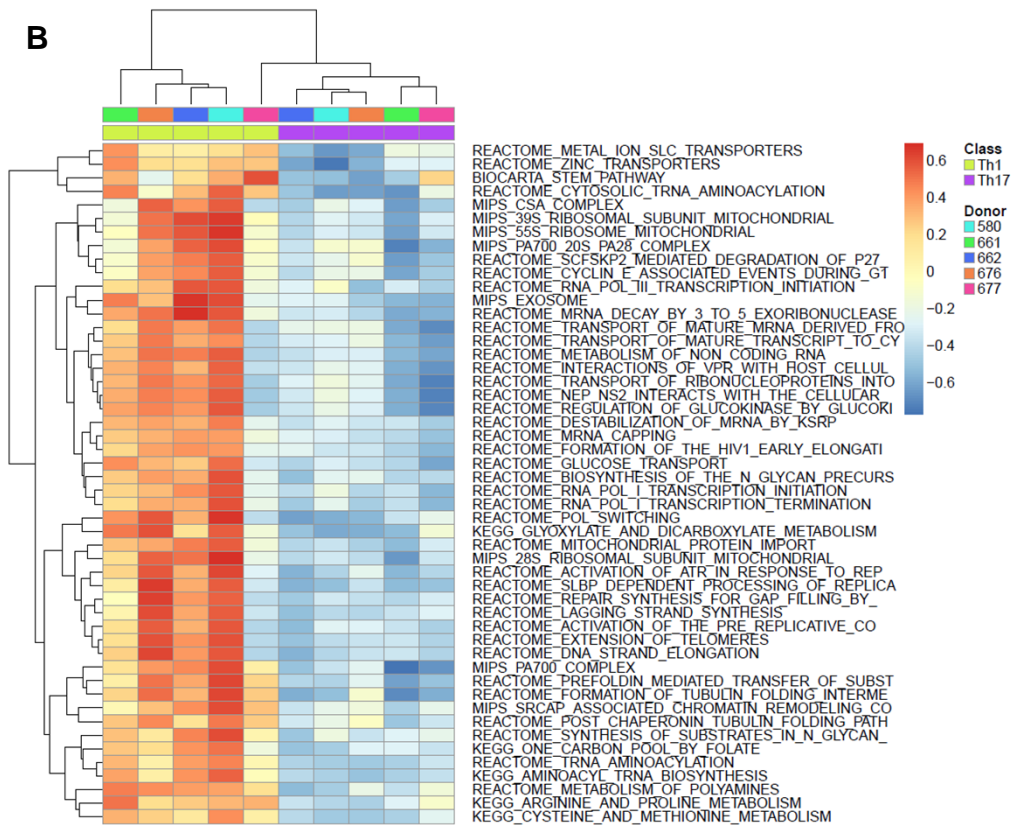

Supplement: Supplementary file 3 — 10.1186/s12977-015-0226-9 a A heat map including the hierarchical clustering of all differentially expressed genes in Th17 versus Th1 subsets and b a heat map including top differentially expressed canonical pathways in Th17 versus Th1 subsets identified using Gene set variation analysis (GSVA; adj. p-values < 0.05). [file 12977_2015_226_MOESM3_ESM.pdf]

## C2. Canonical pathways Th17 vs. Th1

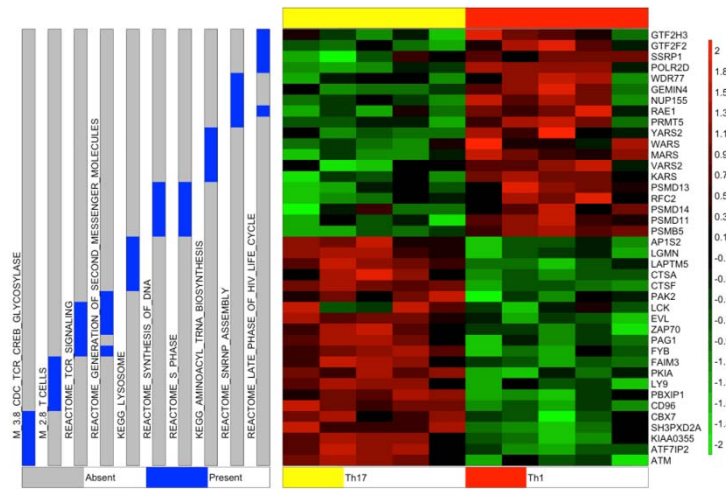

## C3. Transcription factors Th17 vs. Th1

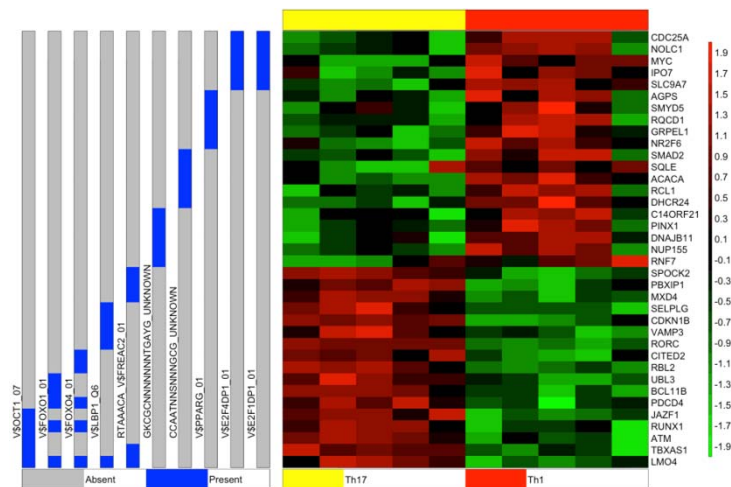

## C5. Biological processes Th17 vs. Th1

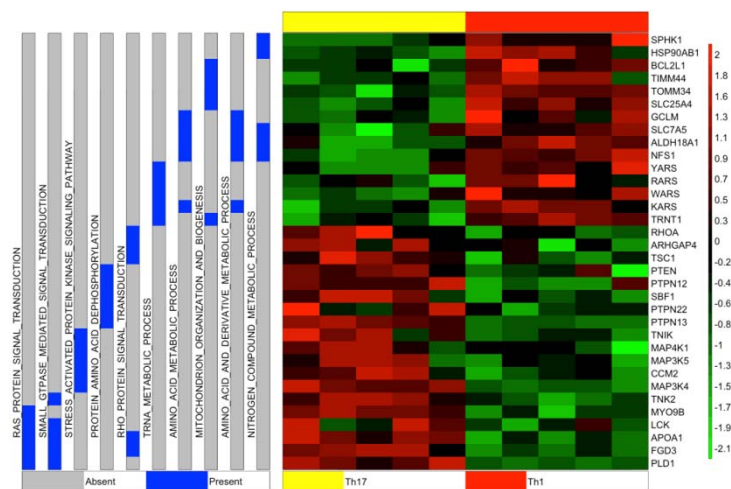

Supplement: Supplementary file 5 — 10.1186/s12977-015-0226-9 Included are top canonical pathways (C2) (a), transcription factors (C3) (b), and biological processes (C5) (c) that are differentially expressed between Th17 and Th1 subsets and were identified using Gene set enrichment analysis (GSEA). [file 12977_2015_226_MOESM5_ESM.pdf]

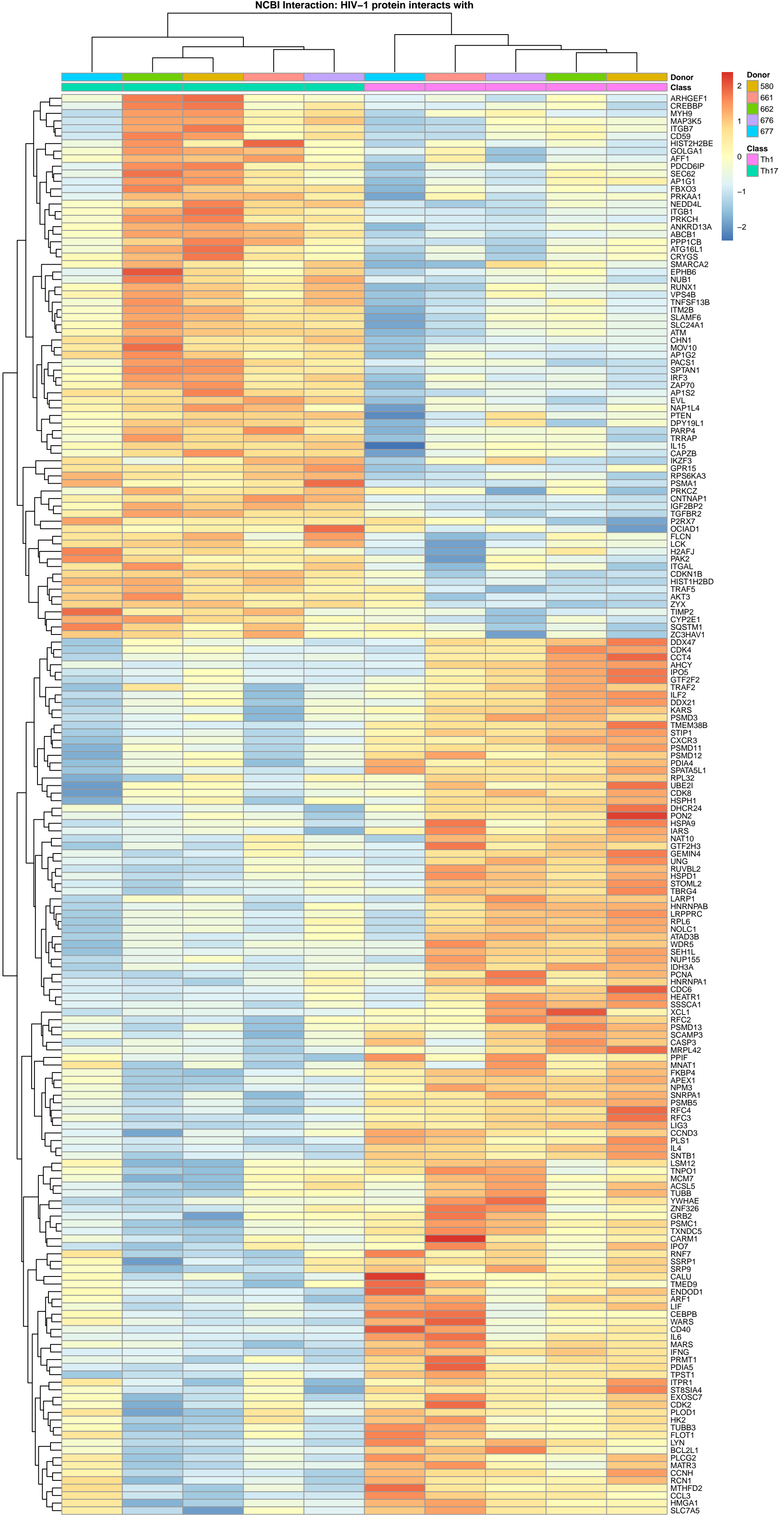

Supplement: Supplementary file 6 — 10.1186/s12977-015-0226-9 A meta-analysis for transcripts included in the NCBI HIV Interaction data base that were identified as top differentially expressed between Th17 and Th1 subsets. [file 12977_2015_226_MOESM6_ESM.pdf]

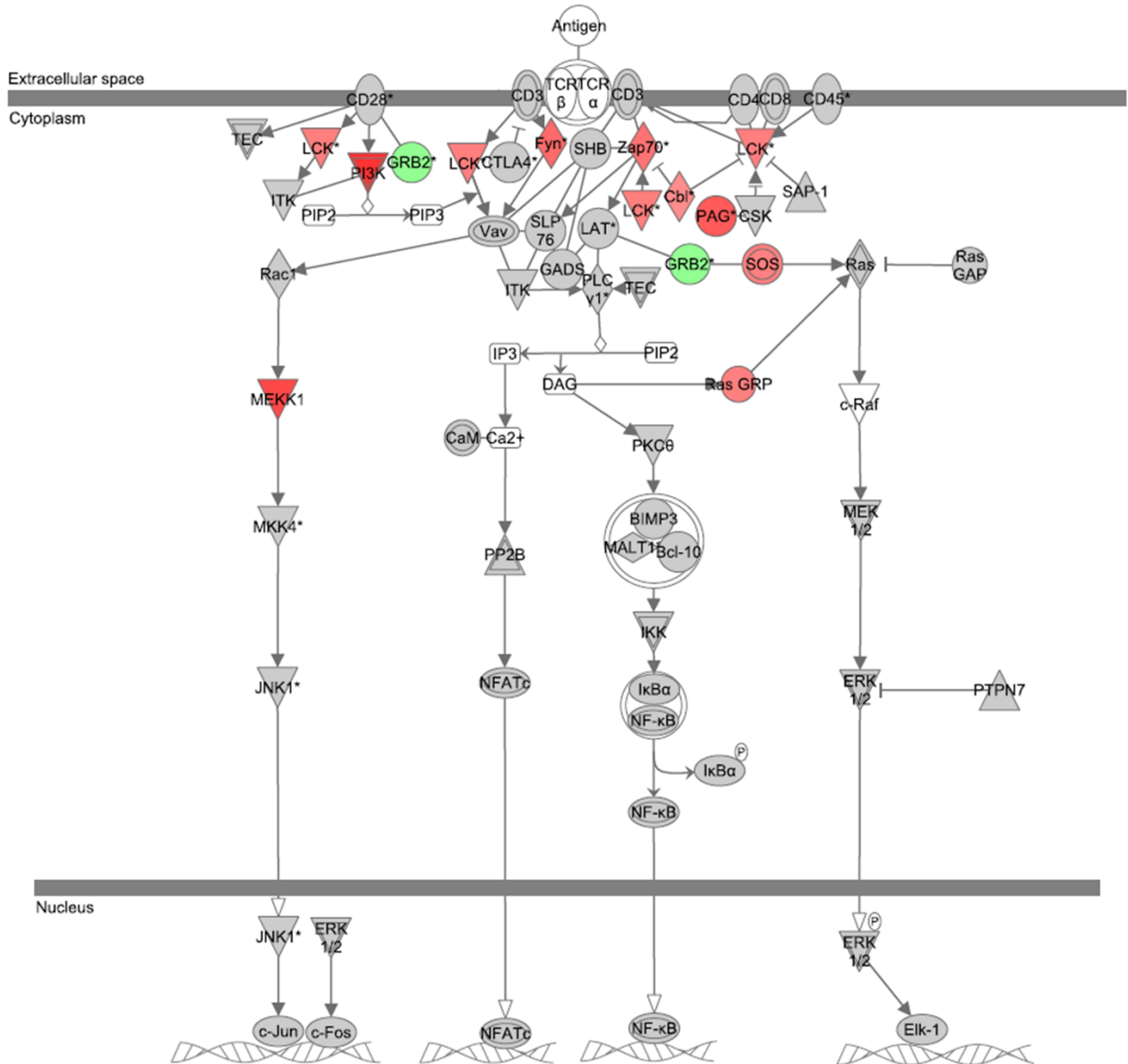

Supplement: Supplementary file 7 — 10.1186/s12977-015-0226-9 The T cell receptor signalling pathways was generated using the Ingenuity pathway analysis (IPA), with transcripts up and down regulated in Th17 versus Th1 subsets being highlighted in red and green, respectively. [file 12977_2015_226_MOESM7_ESM.pdf]
